# Comparative Analysis of the TRB Locus in the *Camelus* Genus

**DOI:** 10.3389/fgene.2019.00482

**Published:** 2019-05-24

**Authors:** Rachele Antonacci, Mariagrazia Bellini, Giovanna Linguiti, Salvatrice Ciccarese, Serafina Massari

**Affiliations:** ^1^Department of Biology, University of Bari Aldo Moro, Bari, Italy; ^2^Department of Biological and Environmental Sciences and Technologies, University of Salento, Lecce, Italy

**Keywords:** T cell receptor, TRB locus, Old World camelids, ImMunoGeneTics database, TRY genes

## Abstract

T cells can be separated into two major subsets based on the heterodimer that forms their T cell receptors. αβ T cells have receptors consisting of α and β chains, while γδ T cells are composed of γ and δ chains. αβ T cells play an essential role within the adaptive immune responses against pathogens. The recent genomic characterization of the *Camelus dromedarius* T cell receptor β (TRB) locus has allowed us to infer the structure of this locus from the draft genome sequences of its wild and domestic Bactrian congeners, *Camelus ferus* and *Camelus bactrianus*. The general structural organization of the wild and domestic Bactrian TRB locus is similar to that of the dromedary, with a pool of TRBV genes positioned at the 5′ end of D-J-C clusters, followed by a single TRBV gene located at the 3′ end with an inverted transcriptional orientation. Despite the fragmented nature of the assemblies, comparative genomics reveals the existence of a perfect co-linearity between the three Old World camel TRB genomic sequences, which enables the transfer of information from one sequence to another and the filling of gaps in the genomic sequences. A virtual camelid TRB locus is hypothesized with the presence of 33 TRBV genes distributed in 26 subgroups. Likewise, in the artiodactyl species, three in-tandem D-J-C clusters, each composed of one TRBD gene, six or seven TRBJ genes, and one TRBC gene, are placed at the 3′ end of the locus. As reported in the ruminant species, a group of four functional TRY genes at the 5′ end and only one gene at the 3′ end, complete the camelid TRB locus. Although the gene content is similar, differences are observed in the TRBV functional repertoire, and genes that are functional in one species are pseudogenes in the other species. Hence, variations in the functional repertoire between dromedary, wild and domestic Bactrian camels, rather than differences in the gene content, may represent the molecular basis explaining the disparity in the TRB repertoire between the *Camelus* species. Finally, our data contribute to the knowledge about the evolutionary history of Old World camelids.

## Introduction

Old World camelids consist of three extant species, including the one-humped camel or dromedary, *Camelus dromedarius*, and the two-humped wild and domestic Bactrian camels, *Camelus ferus* and *Camelus bactrianus*. All the species are adapted to live in specific weather conditions of both desert and semi-desert regions and undergo environmental pressures, potentially resulting in the evolution of adaptations specific to each species. While the species status of the dromedary and domestic Bactrian camel has been established, the evolutionary relationship between the two-humped camels has long been debated, and only recently the wild two-humped camel has been recognized as a separate species, *Camelus ferus*, on the basis of the mitochondrial (Ji et al., 2009; Silbermayr et al., 2010; Mohandesan et al., 2017) and nuclear (Jirimutu et al., 2012; Silbermayr and Burger, 2012) genetic data.

The camelid species are an interesting model to study the immune system responsible for antigen recognition (Ciccarese et al., unpublished). Together with B cells, which produce immunoglobulin (IG), αβ and γδ T cells are the major cellular components of the adaptive immune system. A specialized genetic machinery, from simple interchanges of a small number of genes, creates great diversity in the IG and in αβ and γδ T cell receptor (TR) that potentially generate billions of different IG and TR. The adaptive immune response of camels displays characteristic features, such as heavy chain antibody homodimers in the serum (Hamers-Casterman et al., 1993; Muyldermans et al., 2009) and a limited germline repertoire of T cell receptor γ (TRG) and δ (TRD) chain genes compared to other artiodactyl species (Vaccarelli et al., 2008; Piccinni et al., 2015), diversified by the extensive somatic hypermutation (SMH) (Antonacci et al., 2011; Vaccarelli et al., 2012; Ciccarese et al., 2014). In the same way, the *Camelus dromedarius* repertoire of T cell receptor β (TRB) chain appears reduced in size and gene content compared to the other species (Antonacci et al., 2017a,b). Anyhow, the structural organization of the *Camelus dromedarius* TRB locus is similar to that of the other mammalian species, with a pool of Variable (TRBV) genes positioned at the 5′ end of Diversity (TRBD), Joining (TRBJ) and Constant (TRBC) genes, followed by a single TRBV gene, with an inverted transcriptional orientation located at the 3′ end. The TRBD, TRBJ and TRBC genes are organized in three D-J-C clusters, which is a common feature of sheep, cattle and pigs.

To broaden the understanding of the camel immune system, we characterized the structure of the TRB locus in *Camelus ferus* and *Camelus bactrianus* analyzing the draft genome sequences available in public database, using the human (Lefranc et al., 2003) and dromedary TRB locus (Antonacci et al., 2017a,b) as reference sequences.

## Materials and Methods

### Genome Analyses

The *Camelus ferus* (wild Bactrian camel) TRB genomic sequence was retrieved directly from the CB1 assembly of the whole genome shotgun sequence available at GenBank (BioProject PRJNA76177). In particular, the whole TRB region of 302258 bp (gaps included) is entirely contained in the NW_006210980 unplaced genomic scaffold. The MOXD2 (monooxygenase, dopamine-beta-hydroxylase-like 2, DBH-like2) and EPHB6 (ephrin type-B receptor 6) genes, flanking, respectively, the 5′ and 3′ ends of TRB locus, were included in the analysis.

The *Camelus bactrianus* (domestic Bactrian camel) TRB genomic sequence was retrieved from the Ca_bactrianus_MBC_1.0 assembly of the whole genome shotgun sequence available at GenBank (BioProject PRJNA183605). The analyzed region comprises two principal unplaced genomic not continuous scaffolds: NW_011511605 (181382 bp) and NW_011509864 (99628 bp). MOXD2 and EPHB6, included in the analysis, are located within the NW_011511605 and the NW_011509864 scaffolds, respectively. A further BLAST search of the domestic Bactrian camel genomic assembly was performed, using, as a query, specific dromedary TRB gene sequences as well as the 3′ end and the 5′ end sequences of the NW_011511605 and NW_011509864 scaffolds, respectively. In this way, five short unplaced scaffolds were retrieved, including NW_011537499, which is 542 bp long and overlaps with the 3′ end of NW_011511605 and the 5′ end of NW_011509864, two continuous but not overlapping scaffolds, namely, NW_011541550 (877 bp) and NW_011529568 (303 bp), which contain the TRBV15 gene, NW_011514083 (2181 bp), which retains the TRBV16 gene, and NW_011511596 (685 bp) which retains the TRBV21S3 gene. The length of the whole TRB genomic sequence is 285598 bp (gaps included). All the scaffolds retrieved from the genomic assemblies are summarized in Supplementary Table S1.

The human and the dromedary TRB genomic sequences (Lefranc et al., 2015; Antonacci et al., 2017a,b) were used against the *Camelus ferus* and *Camelus bactrianus* genome sequences to identify the corresponding TRBV, TRBD, TRBJ, and TRBC genes based on homology, by the BLAST program.

The beginning and end of each coding exon were identified with accuracy by the presence of splice sites or the flanking recombination signal sequences (RSs) of the TRBV, TRBD and TRBJ genes. The locations of the TRB genes are provided in Supplementary Tables S2, S3. The sequence comparison also allowed for the identification and characterization of the camel trypsin-like serine protease (TRY) genes. The locations of the TRY genes are provided in Supplementary Tables S4, S5.

The PipMaker program (Schwartz et al., 2000)^1^ was also used for the genomic comparative analyses of the *Camelus ferus* and *Camelus bactrianus* TRB loci with the dromedary sequence previously described (Antonacci et al., 2017a,b). Moreover, the computational analysis was conducted using the RepeatMasker for the identification of genome-wide repeats and low complexity regions^1^.

### Classification of the TRB Genes

Considering the percentage of nucleotide identity of the genes with respect to human and *Camelus dromedarius* and based on the genomic position within the locus, each TRB gene was classified, and the nomenclature was established according to IMGT at http://www.imgt.org/IMGTScientificChart/SequenceDescription/IMGTfunctionality.html (Lefranc, 2014; Supplementary Tables S2, S3). The TRBV genes were assigned to 26 different subgroups in *Camelus ferus* as well as in *Camelus bactrianus*, based on the percentage of nucleotide identity by using Clustal Omega alignment tool, which is available at EMBL-EBI website^2^, adopting the criterion that sequences with a nucleotide identity of more than 75% in the V-region belong to the same subgroup (Arden et al., 1995a,b). Due to the fragmented and incomplete nature of the genomic assemblies, a temporary designation was used for multigene subgroups, in which the Arabic number (for the subgroup) is followed by the letter S followed by the number of the gene in the subgroup.

The TRBD, TRBJ, and TRBC genes were annotated, according to the similarity with the D-J-C sequence of the dromedary species (Antonacci et al., 2017a,b). Each TRBJ1, TRBJ2 and TRBJ3 gene was designed by a hyphen and a number corresponding to their position in the cluster. They were all predicted to be functional except for the domestic Bactrian camel TRBJ3-5 and TRBJ2-6 genes whose sequences were incomplete (Supplementary Tables S2, S3).

### Phylogenetic Analyses

The TRBV genes used for the phylogenetic analysis were retrieved from the following sequences deposited in the GEDI (for GenBank/ENA/DDBJ/IMGT/LIGM-DB) databases: NG_001333 (human TRB locus contig); NW_011591622, NW_011593440, NW_011591151, NW_011620189, NW_011616084, NW_011607149, NW_011601111, and LT837971 [dromedary TRB locus contig as characterized by (Antonacci et al., 2017a,b)]; NW_006210980 (this work) (wild Bactrian camel TRB locus contig); and NW_011511605, NW_011509864, NW_011514083 and NW_011511596 (this work) (domestic Bactrian camel TRB locus contig). We combined the nucleotide sequences of the V-REGION of the TRBV genes with the corresponding gene sequences of humans and dromedary.

The TRBC genes used for the phylogenetic analysis were retrieved from the following sequences deposited in the GEDI databases: NG_001333 (human TRB locus); AE000665 (mouse TRB locus); NW_003726086 [dog TRB locus as characterized by Mineccia et al. (2012)]; NW_003159384 [rabbit TRB locus as characterized by Antonacci et al. (2014)]; L27845 and L27844 [horse TRBC1 and TRBC2, Schrenzel et al. (1994)]; AM420900 (sheep D-J-C region, Antonacci et al., 2008); GK000004 [bovine TRB locus as characterized by Connelley et al. (2009)]; NC_010460 [pig TRB locus as characterized by Massari et al. (2018)]; LT837971 (dromedary D-J-C region, Antonacci et al., 2017b); NW_006210980 (this work) (*Camelus ferus* TRB locus); and NW_011509864 (this work) (*Camelus bactrianus* D-J-C region).

For the TRY analysis, only complete and functional genes were used. They were retrieved from the following sequences deposited in the GEDI databases: NG_001333 for three human TRY genes (PRSS58, PRSS1, and PRSS2); four dog TRY genes derived from Mineccia et al., 2012; five rabbit TRY genes derived from Antonacci et al., 2014, renamed from TRY1 to TRY5 according to the order on the TRB locus; five cattle and sheep TRY genes (TRY1-TRY5) derived from Connelley et al., 2009 and from NC_019461 (personal communication), respectively, NC_010460 for four pig genes (TRY1-TRY4 as in Massari et al., 2018); NW_011591622 and NW_011591151 for three dromedary genes (TRY1, TRY2, and TRY4) named on the basis of the bovine nomenclature; NW_006210980 for three *Camelus ferus* genes (this work); and NW_011511605 and NW_011509864 for five *Camelus bactrianus* genes (this work).

The information about genes used in the phylogenetic analyses are summarized in Supplementary Table S6.

Multiple alignments of the gene sequences under analysis were carried out with the MUSCLE program (Edgar, 2004). The evolutionary analyses were conducted in MEGA7 (Kumar et al., 2016). We used the neighbor-joining (NJ) method to reconstruct the phylogenetic tree (Saitou and Nei, 1987). The evolutionary distances were computed using the p-distance method (Nei and Kumar, 2000) and are in the units of the number of base differences per site.

## Results

### Genomic Organization of the *Camelus ferus* and *Camelus bactrianus* TRB Loci as Drawn From the Assemblies

A standard BLAST search of the genomic resources was performed by using the human and dromedary TRB gene sequences to assess their location in the wild and domestic Bactrian camel genomes. We directly retrieved a sequence of 302258 bp (gaps included) from the *Camelus ferus* CB1 assembly, which corresponds to one unplaced genomic scaffold, and a sequence of 285598 bp (gaps included) from the *Camelus bactrianus* Ca_bactrianus_MBC_1.0 assembly, which corresponds to seven distinct unplaced scaffolds. In particular, NW_011511605, NW_011509864 and NW_011537499 are overlapping scaffolds and form a continuous sequence. The genes MOXD2 and EPHB6, which flank the 5′ and 3′ ends, respectively, of all the mammalian TRB loci studied to date, were comprised in the sequences. We identified and annotated all the TRB genes, taking into account, as a reference, both the human and the dromedary sequences^3^, Lefranc et al., 2015; Antonacci et al., 2017a,b). The functionality of the V, D, J and C genes was predicted through the manual alignment of the sequences adopting the following parameters: (a) the identification of the leader sequence at the 5′ of the TRBV genes; (b) the determination of the proper RSs located at 3′ of the TRBV (V-RS) gene, the 5′ and 3′ ends of the TRBD (5′D-RS and 3′D-RS) and the 5′ of the TRBJ (J-RS) gene; (c) the determination of conserved acceptor and donor splicing sites; (d) the estimation of the expected length of the coding regions; and (e) the absence of frameshifts and stop codons in the coding regions of the genes.

**FIGURE 1:**
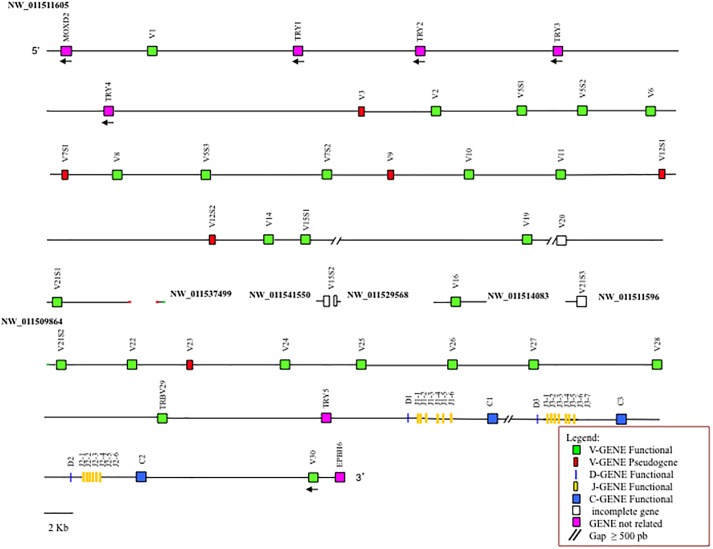
Schematic representation of the genomic organization of the *Camelus bactrianus* TRB locus deduced from the genome assemblies. The diagram shows the position of all the related and unrelated TRB genes according to the nomenclature. The NW_011511605, NW_011537499 and NW_011509864 are overlapping scaffolds, with NW_011537499 in between NW_011511605 and NW_011509864. The boxes representing the genes are not to scale. The white boxes are incomplete genes. The exons are not shown. The arrows indicate the transcriptional orientation of the genes.

The general structural organization of the *Camelus ferus* and *Camelus bactrianus* TRB loci follows that of other mammalian species, with a library of TRBV genes positioned at the 5′ end of the D-J-C clusters, followed by a single TRBV gene, with an inverted transcriptional orientation located at the 3′ end (Figure 1 and Supplementary Figure S1).

It is noteworthy that the analysis of the sequence revealed the presence of only one D-J-C cluster in *Camelus ferus* (Supplementary Figure S1), whereas three D-J-C clusters are present in the *Camelus bactrianus* sequence (Figure 1), as in *Camelus dromedarius* (Antonacci et al., 2017a,b). The fragmented and incomplete nature of the genomic assemblies might justify this discrepancy in the *Camelus ferus* genome.

In *Camelus bactrianus*, the NW_011541550, NW_011529568, NW_011514083, and NW_011511596 scaffolds can be tentatively positioned within the TRB locus, based on the *Camelus dromedarius* as well as the *Camelus ferus* sequence structures.

### Classification of the *Camelus ferus* and *Camelus bactrianus* TRBV Genes and Comparison With the Dromedary Genes

We annotated 30 TRBV germline genes in the wild Bactrian camel genome, while as in dromedary (Antonacci et al., 2017a,b), 33 TRBV genes were found in the domestic Bactrian camel TRB locus. They were assigned to 26 distinct subgroups both in *Camelus ferus* and *Camelus bactrianus.*

**FIGURE 2:**
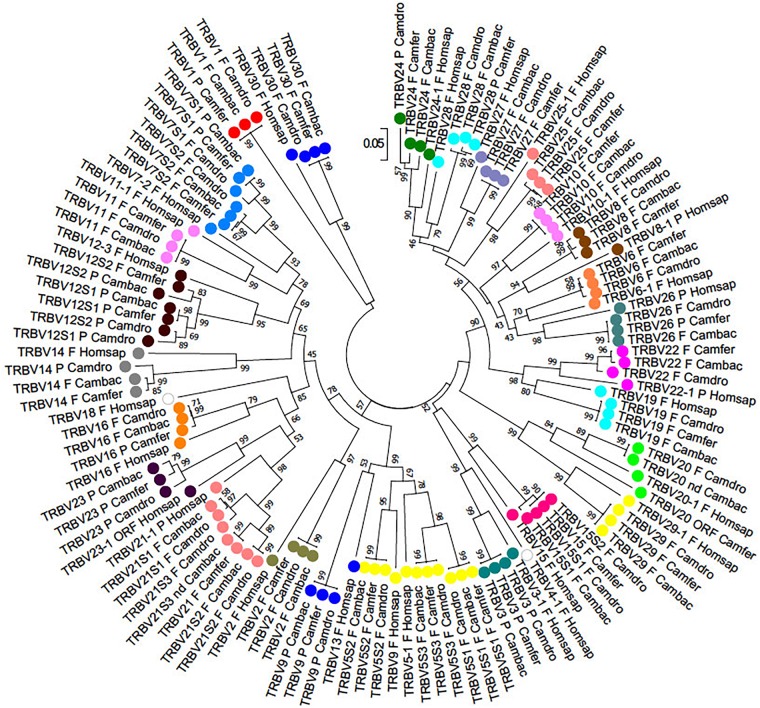
The NJ tree inferred from the wild and domestic Bactrian camel, human and dromedary TRBV gene sequences. The evolutionary analysis was conducted in MEGA7 (Kumar et al., 2016). The optimal tree with the sum of branch length = 8.80179972 is shown. The percentage of replicate trees in which the associated taxa clustered together in the bootstrap test (100 replicates) is shown next to the branches (Felsenstein, 1985). The tree is drawn to scale with branch lengths in the same units as those of the evolutionary distances used to infer phylogenetic trees. The evolutionary distances were computed using the p-distance method (Nei and Kumar, 2000) and are in the units of the number of base differences per site. The analysis involved 123 nucleotide sequences. Codon positions included were 1st+2nd+3rd+Noncoding. All positions containing gaps and missing data were eliminated. There were a total of 130 positions in the final dataset. The different colors highlight the distribution of the phylogenetic groups. The gene functionality according to IMGT rules (F, functional, ORF, open reading frame, P, pseudogene) is indicated; “nd” indicates that the nucleotide sequence of the gene is incomplete and its functionality cannot be defined. The IMGT 6-letter for species (Homsap, Camdro, Camfer, Cambac) standardized abbreviation for taxon is used.

To classify the TRBV gene subgroups, the evolutionary relationship of these genes was investigated by comparing all the wild and domestic Bactrian camel genes with the available genes corresponding to humans and dromedary by adopting two selection criteria as follows: (1) only the potential functional genes and in-frame pseudogenes (except for human TRBV1) were included, and (2) only one gene for each of the human subgroups was selected. Thus, the V-REGION nucleotide sequences of all the selected TRBV genes were combined in the same alignment, and an unrooted phylogenetic tree was made using the NJ method (Saitou and Nei, 1987; Figure 2). The tree shows that each of the *Camelus ferus* as well as of the *Camelus bactrianus* subgroups come together and form a monophyletic group, if present, with a corresponding human and dromedary gene. Therefore, according to phylogenetic clustering, we classified each Bactrian camel TRBV subgroup as orthologous to their corresponding human and dromedary subgroups. The only exception is the *Camelus ferus* and *Camelus bactrianus* TRBV9 genes, which were named as dromedary genes based on their genomic position within the TRB locus (Antonacci et al., 2017a,b), even if they are related to the human TRBV13 gene. Moreover, as already reported (Massari et al., 2018), the human TRBV9 is grouped together with the orthologous TRBV5S2 gene of the other mammalian species. Three human TRBV subgroups (TRBV4, TRBV17, and TRBV18) are lacking in all three camel species, indicating that these subgroups have been lost in these species or alternatively they might have originated after the separation of Camelidae from the other mammalian species. Finally, all the camel TRBV1 genes group together. Table 1 summarizes the correspondence of each TRBV gene subgroup between the camel species, with respect to the humans.

**Table 1:** Correspondence between camel and human TRBV subgroup genes.

Subgroups	Human	*C. dromedarius*	*C. ferus*	*C. bactrianus*
TRBV1	1 (P)	1	1 (P)	1
TRBV2	1	1	1	1
TRBV3	2	1 (P)	1 (P)	1 (P)
TRBV4	3	–	–	–
TRBV5	8	3	3	3
TRBV6	9	1	1	1
TRBV7	9	2	1 (P) + 1	1 (P) + 1
TRBV8	2	1	1	1
TRBV9	1	(TRBV5S2)	(TRBV5S2)	(TRBV5S2)
TRBV10	3	1	1	1
TRBV11	3	1	1	1
TRBV12	5	2 (P)	1 (P)+ 1	2 (P)
TRBV13	1	1 (TRBV9 P)	1 (TRBV9 P)	1 (TRBV9 P)
TRBV14	1	1 (P)	1	1
TRBV15	1	2	1	1 + 1 (nd)
TRBV16	1	1	1 (P)	1
TRBV17	1 (ORF)	–	–	–
TRBV18	1	–	–	–
TRBV19	1	1	1	1
TRBV20	1	1	1 (ORF)	1 (nd)
TRBV21	1	2 + 1 (P)	1	2 + 1 (P)
TRBV22	1	1	1	1
TRBV23	1	1 (P)	1 (P)	1 (P)
TRBV24	1	1 (P)	1	1
TRBV25	1	1	1	1
TRBV26	1	1	1 (P)	1
TRBV27	1	1	1	1
TRBV28	1	1	1 (P)	1
TRBV29	1	1	1	1
TRBV30	1	1	1	1
**TOTAL**	**66**	**33**	**30**	**33**


*The functionality is also reported. P, pseudogene; ORF, open reading frame; Nd, not defined (indicates that the nt sequence of the gene is incomplete and its functionality cannot be defined).*

The TRBV gene functionality was defined based on the IMGT rules as described above. Twenty-one genes in *Camelus ferus* (70%) and twenty-four genes in *Camelus bactrianus* (80%) were predicted to be functional (Supplementary Tables S2, S3, S7). Three subgroups (TRBV5, TRBV7, and TRBV12) in the wild and four subgroups (TRBV5, TRBV7, TRBV12, and TRBV21) in the domestic Bactrian camels are multimembers, with a limited number of genes (from 2 to 3). The TRBV20 gene has an anomalous V-EXON but with an open reading frame in *Camelus ferus*, while the same gene is incomplete in *Camelus bactrianus.* The *Camelus bactrianus* TRBV21S3 gene is also incomplete. Moreover, the TRBV15S2 gene is split in the NW_011541550 and NW_011529568 scaffolds, and because of a gap in between, it lacks a portion of the V-EXON.

Nine TRBV genes in *Camelus ferus* and six in *Camelus bactrianus* are pseudogenes (Supplementary Table S7). Five of these (TRBV3, TRBV7S1, TRBV9, TRBV12S1, and TRBV23) are shared between the two loci. TRBV3, TRBV9, and TRBV23 are also pseudogenes in dromedary (Antonacci et al., 2017a), while the TRBV7S1 gene is functional. Moreover, the TRBV14 and TRBV24 genes are pseudogenes in dromedary, whereas they are functional in both the wild and domestic Bactrian camel. Four TRBV pseudogenes (TRBV1, TRBV16, TRBV26 and TRBV28) in *Camelus ferus* are functional in *Camelus bactrianus* and *Camelus dromedarius.* In contrast, the TRBV12S1 gene is functional in *Camelus ferus*, but is a pseudogene in *Camelus bactrianus* and *Camelus dromedarius.* We must consider that some of these discrepancies might be due to sequence errors.

The deduced amino acid sequences of the wild and domestic Bactrian camel germline TRBV genes were manually aligned together with the corresponding dromedary genes according to IMGT unique numbering for the V-REGION (Lefranc et al., 2003) to maximize the percentage of identity (Supplementary Figure S2). A great sequence identity between orthologous genes was observed, confirming the close relatedness of the three camel species. Mostly, the amino acid variations might be ascribed to allelic polymorphisms. Few amino acid differences (more than two residues) characterized the TRBV6, TRBV7S1, TRBV8, TRBV24 and TRBV27 genes.

### Characterization of the D-J-C Region

In *Camelus ferus*, only one D-J-C cluster was detected, with one TRBD gene, seven TRBJ genes and one TRBC gene (Supplementary Figure S1). The nucleotide sequence comparison with the dromedary TRB genes (Antonacci et al., 2017a) revealed that the TRBD and the TRBJ genes are homologous to the corresponding genes of the D-J-C cluster 1, whereas the TRBC gene corresponds to the dromedary TRBC2 gene. Although a different organization of the D-J-C region in wild camel, with respect to the dromedary, cannot be excluded, the discrepancy is most likely due to a gap in the genomic assembly that comprises the TRBC1 gene, the entire D-J-C cluster 3, the TRBD2 and the TRBJ2 genes. This conclusion is in accordance with the presence of three D-J-C clusters (Figure 1) in *Camelus bactrianus* that perfectly match the structure of the *Camelus dromedarius* TRB locus except for the lack of the TRBJ3-6 gene, which is likely due to the gap present in the genomic assembly.

Moreover, the TRBJ1 cluster in *Camelus ferus* consists of seven genes, one more than in *Camelus dromedarius* as well as in *Camelus bactrianus*. The sequence analysis showed that the region of 315 bp, containing the TRBJ1-7 gene (from position 3052198 to 3052512 of NW_006210980) is identical to the TRBJ1-4 region. This redundancy indicates a probable error in the sequence assembly whereby the TRBJ1-7 gene should be excluded from the *Camelus ferus* TRB locus.

All the genes were analyzed in detail for their structure (Supplementary Figure S3) and functionality (Supplementary Tables S2, S3). The only exceptions are the *Camelus bactrianus* TRBJ2-6, TRB3-5 and TRBC1 genes whose functionality cannot be defined for the incomplete nucleotide sequence (Supplementary Figures S3b,c). The comparison between the wild and domestic Bactrian camels showed that the sequences of the orthologous genes are identical, except for the TRBJ1-6 gene, which is present in *Camelus bactrianus* with un unusual amino acid FGXG motif (FGLS), and for a polymorphism in the first exon of the TRBC2 gene (Supplementary Figures S3b,c).

The comparison with the dromedary genes (Antonacci et al., 2017a,b) revealed a perfect correspondence between orthologous genes; the only exception is the possible exchange between the TRBD2 and TRBD3 genes in *Camelus bactrianus*, with respect to the reference genomic sequence of *Camelus dromedarius.*

In Supplementary Figure S3c, the protein display of the wild and domestic Bactrian camel TRBC genes, compared with the reference sequences of the dromedary genes, is shown. All the TRBC genes encode a similar protein of 178 amino acids, with the extracellular domain encoded by exon 1, exon 2 and by the first codon of exon 3. The transmembrane region is encoded by the remaining part of exon 3, while the cytoplasmic portion is encoded by exon 4.

**FIGURE 3:**
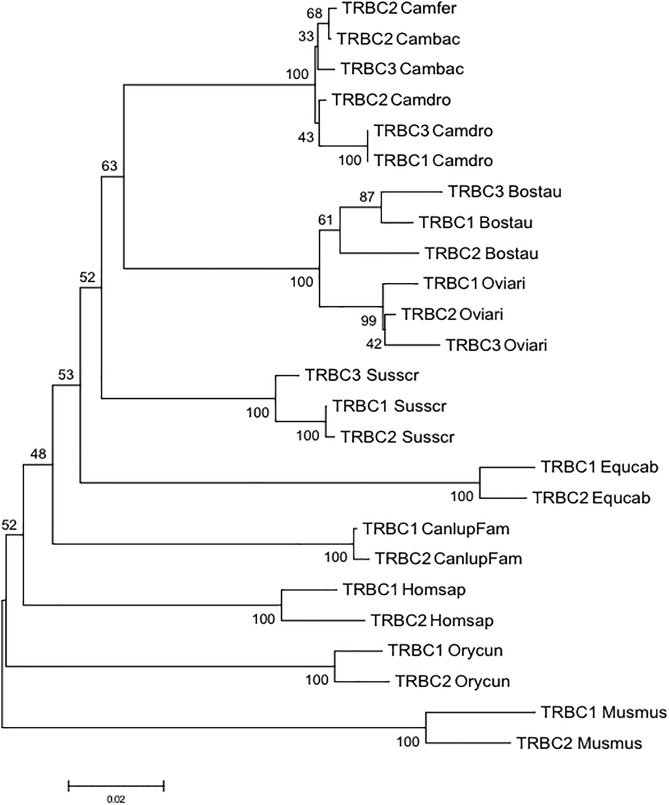
The NJ tree inferred from the TRBC gene sequences within mammalian species. The evolutionary analysis was conducted in MEGA7 (Kumar et al., 2016). The percentage of replicate trees in which the associated taxa clustered together in the bootstrap test (100 replicates) is shown next to the branches (Felsenstein, 1985). The tree is drawn to scale with branch lengths in the same units as those of the evolutionary distances used to infer phylogenetic trees. The evolutionary distances were computed using the p-distance method (Nei and Kumar, 2000) and are in the units of the number of base differences per site. The analysis involved 25 nucleotide sequences. Codon positions included were 1st+2nd+3rd+Noncoding. All positions containing gaps and missing data were eliminated. There were a total of 515 positions in the final dataset. The IMGT 6-letter for species (Homsap, Musmus, Susscr, Bostau, Oviari, Equcab, Orycun, Camdro, Camfer, and Cambac) and 9-letter for subspecies (Canlupfam) standardized abbreviation for taxon is used.

To gain insight into the evolution of the camel TRBC genes within Camelidae family and with respect to Artiodactyla, we combined in the same alignment the nucleotide sequences of the coding regions of the *Camelus ferus, Camelus bactrianus* and *Camelus dromedarius* TRBC genes together with those derived from cow, sheep and pig. The TRBC sequences retrieved from the human, mouse, dog, horse and rabbit TRB loci were also included in the analysis. A phylogenetic tree was constructed using the NJ method (Figure 3). In the tree, different from the TRBV genes, the TRBC genes are grouped in a species-specific manner, indicating a closer relationship between paralogous TRBC genes rather than orthologous genes. The only exception seems to be the tight vicinity between the wild and domestic Bactrian camel TRBC2 genes. Moreover, within Artiodactyla, the camel TRBC genes form a sister group with the ruminant genes, whereas the pig genes represent a paraphyletic taxon.

### Phylogenetic Analysis of the TRY Genes

The comparison of the entire wild and domestic Bactrian camel sequences to the dromedary and human sequences allowed us also to identify and annotate unrelated TRB genes, consisting of the MOXD2 and EPHB6 genes, which delimit the TRB locus, and of a group of TRY genes that are typically interspersed among mammalian TRB genes.

Downstream of the TRBV1 gene, proceeding from 5′ to 3′, we found two in *Camelus ferus* and four TRY genes in *Camelus bactrianus* (Figure 1 and Supplementary Figure S1). In both genomic sequences, a further TRY gene was found before the D-J-C region.

**FIGURE 4:**
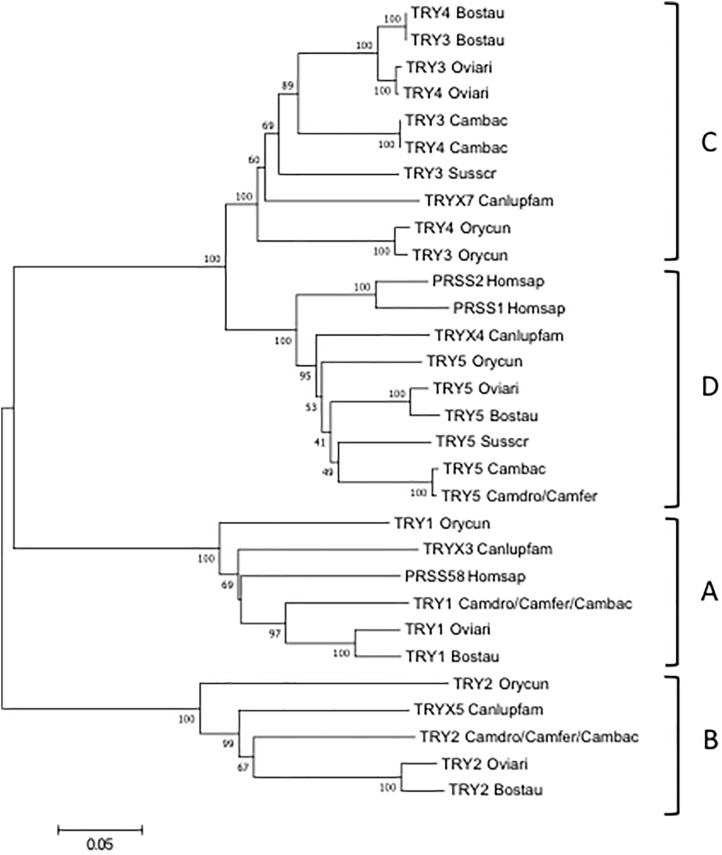
The NJ tree inferred from the TRY gene sequences. The evolutionary analysis was conducted in MEGA7 (Kumar et al., 2016). The optimal tree with the sum of branch length = 1.55315431 is shown. The percentage of replicate trees in which the associated taxa clustered together in the bootstrap test (100 replicates) are shown next to the branches (Felsenstein, 1985). The tree is drawn to scale, with branch lengths in the same units as those of the evolutionary distances used to infer the phylogenetic tree. The evolutionary distances were computed using the p-distance method (Nei and Kumar, 2000) and are in the units of the number of base differences per site. The analysis involved 21 nucleotide sequences. Codon positions included were 1st+2nd+3rd+Noncoding. All positions containing gaps and missing data were eliminated. There were a total of 693 positions in the final dataset. The IMGT 6-letter for species (Homsap, Susscr, Bostau, Oviari, Camdro, Camfer, and Cambac) standardized abbreviation for taxon is used.

To classify the camel TRY, the wild and domestic Bactrian gene sequences were aligned with those of human, dog, rabbit, dromedary, pig, cow and sheep and an phylogenetic tree was made (Figure 4).

The tree resolves the TRY genes in four monophyletic groups, each containing a *Camelus ferus* and/or *Camelus bactrianus* gene. Similar to the TRBV analysis, each group consists of orthologous TRY genes in accordance with the genomic location within the TRB locus. In A, B and C groups are present the TRY genes positioned at the 5′ region, whereas in D the TRY genes located before the D-J-C region. It is noteworthy that in C, the TRY3 and TRY4 genes of ruminants as well as of *Camelus bactrianus* are clustered in a specie-specific manner, similar to the TRBC genes, indicating their occurrence through an independent duplication event in each species or alternatively a process of sequence homogenization of the two genes within each species. A duplication event also occurred in rabbit, generating the TRY3 and TRY4 genes.

The classification, location and predicted functionality of the TRY genes are reported in Supplementary Tables S4, S5.

### Genomic Comparison of the *Camelus* TRB Locus

To trace the genomic architecture of the camelid TRB locus, the TRBV region (from MOXD2 to TRY5) of the three Old World camel species were first screened with the RepeatMasker program to analyse the compositional properties (G + C content) and to identify the interspersed repeats (Supplementary Table S8). The GC content is approximately 45% in all three camel species. The density of the total interspersed repeats is slightly higher in *Camelus ferus* (29.68%) than *Camelus dromedarius* (28.15%) and *Camelus bactrianus* (28.82%). In all cases, the most abundant repeat elements are LINEs, whereas the percentage of SINEs seems to be lower than that in the human and other mammalian corresponding loci (Mineccia et al., 2012; Massari et al., 2018).

Moreover, the *Camelus ferus* and *Camelus bactrianus* sequences were aligned with the dromedary one using the PipMaker program (Schwartz et al., 2000), and the alignment is expressed as a percentage identity plot (pip) (Supplementary Figure S4). The presence in the pip of the superimposed lines indicates the occurrence of redundant matches along the entire region. The clearest matches correspond to all the camel TRBV genes, except for TRBV1, due to the homology among the genes. The horizontal and long continuous lines, which represent ungapped alignments, are evident along the entire region, indicating a co-linearity between the three sequences, with a high percentage of similarity. In contrast, the rare interruptions of the homology line indicate the presence of probable gaps in the corresponding genomic assembly. As an example, there is an interrupted homology line within the domestic Bactrian camel sequence in correspondence of the dromedary TRBV16 gene (Supplementary Figure S4), which might confirm the insertion of the NW_011514083 scaffold within the *Camelus bactrianus* TRB sequence (Figure 1).

## Discussion

The development of new sequencing methodologies has made it possible to explore many mammalian genomes, providing a significant amount of material for large-scale comparative analyses. In general, despite the presence of gaps, due to the incompleteness of genome assemblies, many genes involved in complex biological traits have been discovered and mapped, contributing to the identification of molecular basis of phenotypic differences between species.

In this perspective, the recent publication of the first complete genome sequence of species belonging to the Tylopoda suborder, i.e., dromedary (Wu et al., 2014) and its related domestic and wild Bactrian species (Jirimutu et al., 2012), is a stimulant.

In this paper, we used the recent characterization of the *Camelus dromedarius* TRB locus, encoding for the β chain of the αβ T cell receptor, to establish the genomic organization of this locus in the *Camelus ferus* and *Camelus bactrianus* genomes.

The most interesting aspect that emerges from the analysis of the TRB locus is that, unlike other mammalian species, in which the TRBV genomic region is highly variable in size, in numbers of genes and subgroups as well as in the extent of polymorphisms, this appears very similar among the Old World camelid species. The locus is flanked by the MOXD2 gene at 5′ end and the EPHB6 gene at 3′ end. It consists of a pool of homologous TRBV genes, 30 genes in *Camelus ferus* and 33 in *Camelus bactrianus* as in *Camelus dromedarius*, which, in all cases, were assigned to 26 different subgroups distributed in approximately 240 Kb (Figure 1 and Supplementary Figure S1; Antonacci et al., 2017a,b). Three TRY genes in the wild Bactrian camel, as well as in dromedary, and five genes in the domestic Bactrian camel complete the region.

The phylogenetic analysis of the TRBV genes revealed an inter-species clustering of the orthologous genes with a closer relationship between the camelid species (Figure 2). This allowed for a classification of the TRBV genes in *Camelus ferus* and *Camelus bactrianus*, with respect to *Camelus dromedarius* as well as to humans. Overall, in all the three camelid TRB loci there are three TRBV subgroups (TRBV4, TRBV17 and TRBV18) that are missing compared to human, and only five subgroups (TRBV5, TRBV7, TRBV12, TRBV15 and TRBV21 subgroups) are multimembers, with a limited number of genes (from 2 to 3) (Table 1). Therefore, it is not the number of the TRBV subgroups but rather the absence of extensive duplications within the individual subgroups to make the camelid TRB locus the one with the least number of TRBV germline genes compared to those of the other mammalian species studied thus far (Connelley et al., 2009; Mineccia et al., 2012; Antonacci et al., 2014; Massari et al., 2018). A limited germline TRV number has already been described in the dromedary TRG locus, although a somatic hypermutation mechanism increases the repertoire diversity of the γ and δ chains (Antonacci et al., 2011; Vaccarelli et al., 2012; Ciccarese et al., 2014).

The classification of the TRBV pseudogenes is more articulated, since substantial differences can be observed between the camelid species (Table 1 and Supplementary Table S7). Apart from the TRBV genes classified as pseudogenes in all the three species (TRBV3, TRBV9, TRBV12S1, TRBV21S3 and TRBV23), there are TRBV genes classified as pseudogenes in one species and functional in the other two (TRBV14, TRBV16, TRBV24, TRBV26 and TRBV28). Moreover, in two cases, TRBV7S1 and TRBV12S1, they are pseudogenes in two species but functional in the other. Hence, variations in the functional repertoire, rather than differences in the gene content, represent the molecular basis for the disparity in the TRBV germline repertoire between the Old World camelid species.

Likewise, as with the TRBV genes, the TRY genes are also phylogenetically grouped in an inter-species manner, with the orthologous ones of the other species (Figure 4). Interestingly, a duplication occurred in ruminants (cattle and sheep) and in *Camelus bactrianus* as well as in rabbit (Antonacci et al., 2014), which originated the two very similar TRY3 and TRY4 genes located at the 5′ end of the TRB locus in this species, suggesting that this organization might be shared by herbivorous mammal species. In contrast, the pig and dog TRB loci have a unique corresponding TRY gene, TRY3 and TRYX7, at the 5′ end of the TRB locus (Massari et al., 2018; Mineccia et al., 2012).

**FIGURE 5:**
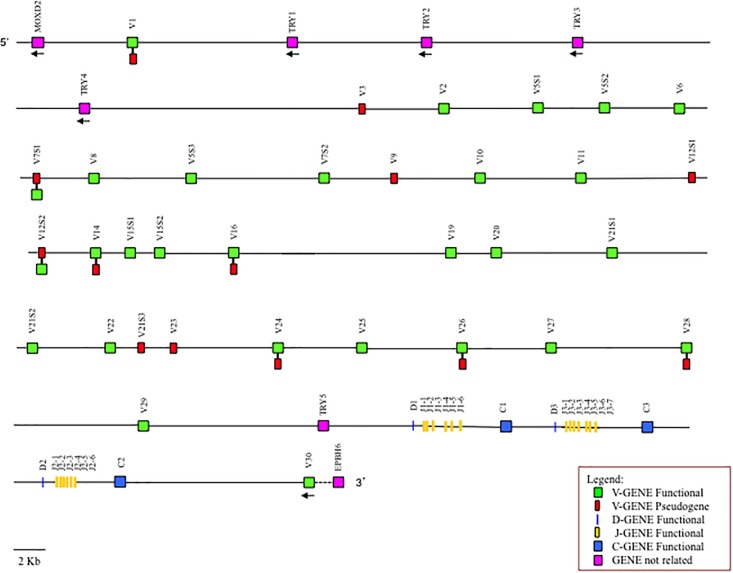
Virtual map of the TRB locus of Old World camelids. The map was constructed according to the indications given in the text. The presence of differences in the functional status of the TRBV genes between the three species is reported.

When we compare the entire TRBV genomic sequences of the Old World camelids, we noted a perfect co-linearity among them, in spite of the fragmented nature of the assemblies, which is distributed throughout the TRB genes as well as the intra-genic regions (Supplementary Figure S4). We exploited this co-linearity to speculate on the genomic organization of the TRB locus in the *Camelus* genus that is able to overcome the gaps present in the individual camelid genomes. Putting together all the collected data, we proposed a virtual map of the TRB locus (Figure 5), adopting the criterion that if a situation is shared by all or at least by two out of the three camelid species it can be considered reliable. Hence, the Old World camelid TRB locus extends over a region of approximately 300 kb. Realistically, 33 TRBV genes, belonging to 26 subgroups, are placed before three in-tandem D-J-C clusters, each composed of one TRBD gene, six or seven TRBJ genes, and one TRBC gene, followed by a single TRBV gene, with an inverted transcriptional orientation located at the 3′ end. According to the tight evolutionary relationship with ruminant genes (Figure 4), five functional TRY genes are presumably interspersed among the TRBV genes, four are situated after the first TRBV gene and one is situated before the D-J-C cluster 1. Differences in the functional aspects of the TRBV genes among the three species were considered and are reported in the map, since they might represent allelic polymorphisms.

## Conclusion

In conclusion, our study highlights the presence of a limited germline TRBV repertoire in *Camelus ferus* and *Camelus bactrianus* as in *Camelus dromedarius* (Antonacci et al., 2017a,b), even though there are wide and diversified TRBD and TRBJ repertoires due the presence of three D-J-C clusters. This is the substantial difference with the closest relatives, i.e., cattle (Connelley et al., 2009) and pig (Massari et al., 2018), where a consistent number of TRBV genes, which occurred by duplications, with a high degree of heterozygosis, precede the three D-J-C clusters.

Altogether, our data, improved the information on the genetic background, allowing to acquire knowledge on the evolutionary history of the Old World camelids.

## Author Contributions

RA, SC, and SM designed research and wrote the manuscript. RA, SM, MB, and GL contributed to the genomic analysis. All authors have read and approved the final manuscript.

## Conflict of Interest Statement

The authors declare that the research was conducted in the absence of any commercial or financial relationships that could be construed as a potential conflict of interest.

## Footnotes

1 http://www.repeatmasker.org

2 http://www.ebi.ac.uk/

3 htpp://www.imgt.org/

## Supplementary Material

FIGURE S1 Schematic representation of the genomic organization of the *Camelus ferus* TRB locus deduced from the genome assemblies. The diagram shows the position of all the related and unrelated TRB genes according to nomenclature. The boxes representing the genes are not to scale. The exons are not shown. The arrows indicate the transcriptional orientation of the genes.

FIGURE S2 The IMGT Protein display of the wild and domestic Bactrian camel together with the dromedary TRBV genes. Only functional genes and in-frame pseudogenes are shown. The description of the strands and loops and of the FR-IMGT and CDR-IMGT is according to the IMGT unique numbering for V-REGION (Lefranc et al., 2003). The five conserved amino acids of the V-DOMAIN (1st-CYS 23, except for the TRBV6 pseudogene, CONSERVED-TRP 41, hydrophobic AA 89, 2nd-CYS 104 and J-PHE 118) are indicated in bold. The amino acid length of the CDR-IMGT is also indicated in square brackets.

FIGURE S3 Nucleotide and deduced amino acid sequences of the *Camelus ferus* and *Camelus bactrianus* TRBD **(a)**, TRBJ **(b)** and TRDC **(c)** genes. The consensus sequence of the heptamer and nonamer is provided at the top of the figure and is underlined. The numbering adopted for the gene classification is reported on the left of each gene. In **(a)**, the nucleotide and deduced amino acid sequences of the TRBD genes in the three coding frames are reported. They consist of a 12 bp (TRBD1), 20 bp (TRBD3) and 15 bp (TRBD2) G-rich stretches. The TRBD1 and TRBD2 genes can be productively read through their three coding phases, whereas the TRBD3 gene can be read only in two phases. The RSs that flank the 5′ and 3′ sides of the coding region are well conserved with respect to the consensus. In **(b)**, the nucleotides and deduced amino acid sequences of all the TRBJ genes are reported. The donor splice site for each TRBJ is shown. The canonical FGXG amino acid motifs, whose presence defines the functional J genes, are underlined. The unusual TRBJ1-6 gene motif is in bold. The TRBJ1-7 redundant gene is in italics. In **(c)**, the IMGT Protein display of the wild, domestic Bactrian and dromedary camel TRBC genes. The descriptions of the strands and loops were collected according to the IMGT unique numbering for the C-DOMAIN (Lefranc et al., 2005). The amino acid differences among the orthologous genes are shaded in gray.

FIGURE S4 Pip of the TRBV region between dromedary, wild and domestic Bactrian camels. The dromedary sequence is shown on the horizontal axis, and the percentage identity to the wild (Camfer) and domestic (Cambac) Bactrian camels are shown on the vertical axis. The position and orientation of all the genes and repetitive sequences are indicated. The horizontal lines represent the ungapped alignments at the percentage similarity corresponding to the scale on the right.

TABLE S1 Scaffolds of the camel genomic assemblies used in the analysis.

TABLE S2 Description of the TRB genes in the *Camelus ferus* genome assembly. The position of all genes and their classification and functionality are reported.

TABLE S3 Description of the TRB genes in the *Camelus bactrianus* genome assembly. The position of all genes and their classification and functionality are reported.

TABLE S4 Description of the unrelated TRB genes in the *Camelus ferus* genome. The position of all genes and their classification are also reported.

TABLE S5 Description of the unrelated TRB genes in the *Camelus bactrianus* genome. The position of all genes and their classification are also reported.

TABLE S6 GEDI ID of the genes used in the phylogenetic analysis.

TABLE S7 Description of *Camfer* and *Cambac* TRBV ORF and pseudogenes

TABLE S8 Output file of the program RepeatMasker. Summary of the repeat content of *Camelus dromedarius*, *Camelus ferus*, and *Camelus bactrianus* TRBV region is shown. The GC levels are also indicated.
