# Supplementary material for: Comparative Analysis of the TRB Locus in the Camelus Genus
Source: Front Genet. 2019 May 24;10:482. doi: 10.3389/fgene.2019.00482 (PMC6558370; doi:10.3389/fgene.2019.00482)
Supplement: FIGURE S1 — Schematic representation of the genomic organization of the Camelus ferus TRB locus deduced from the genome assemblies. The diagram shows the position of all the related and unrelated TRB genes according to nomenclature. The boxes representing the genes are not to scale. The exons are not shown. The arrows indicate the transcriptional orientation of the genes. [file Data_Sheet_1.PDF]

NW_006210980

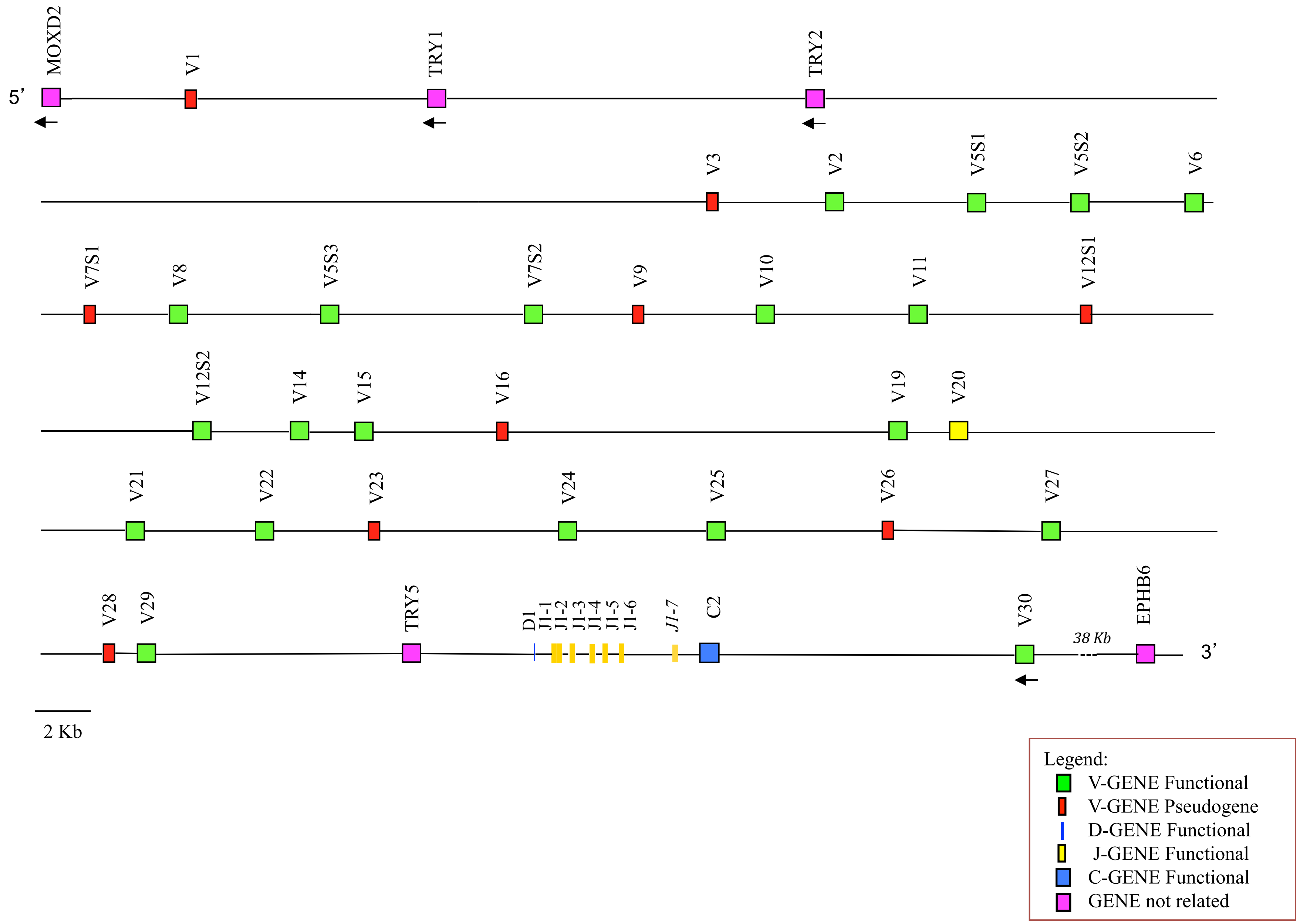

Supplementary Figure S1

Supplementary Figure S2

```
                                                        FR1-IMGT             CDR1-IMGT        FR2-IMGT         CDR2-IMGT                FR3-IMGT                              CDR3-IMGT
                         L-PART1+L-PART2                 (1-26)              (27-38)          (39-55)          (56-65)                  (66-104)                              (105-117)
                                                 A                  B          BC            C          C'          C'C"        C"          D           E            F            FG
                                              (1-15)             (16-26)    (27-38)       (39-46)    (47-55)      (56-65)     (66-74)    (75-84)     (85-96)      (97-104)     (105-117)
                                            ---------------->  ----------->               -------->  --------->               --------->  --------->  ----------->  -------->
Gene       Functionality                    1        10   15  16     23 26 27          38 3941   46 47       55 56        65 66      74 75   80 84 85 89     96 97   104 105
                                            |........|....|   |......|..|  |...........|  |.|....|  |.......|  |.........|  |.......|  |....|...|  |...|.......|  |......|  |....
TRBV1d/b     F     MVLFLILWGWVLMAAVSTDAT   ASFVEQRPRWVLVPR GQAETLRCILR NSQ......YPW MSWYQQDL QGQLQMLAS LRS....PGD KEVISLP.G ADYQATRVNN TELRLHVANVTQ GRTLLCTC SK....[6.6.2]
TRBV1f       P     TVLFLILWGWVLMAAVSTDAT   ASFVEQRPRWVLVPR GQAETLRCILR NSQ......YPW MSWYQQDL QGQLQMLAS LRS....PGD KEVISLP.G ADYQATRVNN TELRLHVANVTQ GRTLLCTC SK....[6.6.2]

TRBV2d       F       MHSRLFCWVIFGLLGVGHT   ELGVSQSPRHYVTAM GQNVTLRCDPI PGH.......LY LSWYRQTP GKGMEFLLS FYN....KAP SEKADFLKD HFLAEMP.NG LYLTLKIQPVQP GDSAMILC ASSV..[5.6.4]
TRBV2f/b     F       MHSRLFCWVIFGLLGVGHT   ELGVSQSPRHYVTAM GQNVTLRCDPI PGH.......LY LSWYRQTP GKGMEFLLS FYN....KAP SEKADFLKD HFSAEMP.NG LYLTLKIQPVQP GDSAMILC ASSV..[5.6.4]

TRBV3d/f/b   P       MGSRLLCCVVLYFLGAGAL   DTGVFQMPKYLITQM GSKMSLKCEQK LGY.......DS MYWYKQGS KQLLKIMFI YNN....KEL ILNETVP.S RFSPEST.DK AHLNLHVDSLEP DDSAMYFC ASS*..[5.6.4]

TRBV5S1d/f/b F       MGSCLLCCVVLCLLGAGPV   DSGVTQTPRHLIKAR GQQVTLSCSPI SGH.......TS VSWYQQAP SEGPQFLFE FYE....KMQ RAKGNFP.D RFSGQQF.DN YSSELIVNFLEV TDSALYFC ASSL..[5.6.4]
TRBV5S2d/f/b F       MGSRLLCWVALCLLGAGQV   DSEVTQTPKYLIKPR KQQVTLRCSPV SGH.......LS VYWYQQAL GQGPQFLIQ YYR....QKE TERGNIS.R RFSGQQF.DN SSSELNVSSLEL TDSALYLC ASSR..[5.6.4]
TRBV5S3d/f/b F       MGPTLLPWAMLCLLGAGPL   EAGVTQTPRHLIKTT GQMVTLRCSPI SGH.......LG ISWYQQAW SQGPQLLFE FYE....RRQ TAKGNFP.D RFLAQQF.GD SRSELNVSSLQL SDSALYLC ASSL..[5.6.4]

TRBV6d       F       MCTCLLCCVAFCLLQAGPV   NAGVIQTPKFQVLKM GQGVTLKCAQD LKH.......DY MYWYRQDP GHGLRLIHY SAG....SGT TNKGDIP.D GYSVSRS.DV ENFTLKLEYATP NQTSVYLC ASSY..[5.6.4]
TRBV6f       F       MCTCLLCCVAFCLLQAGPV   NAGVIQTPKFQVLKM GQGVTLKCAQD LKH.......NY MYWYRQDP GHGLRLIHY SAG....SGT TDKGDIP.D GYSVSRS.DV ENFTLILESATL NQTSVYLC ASKLE.[5.6.5]
TRBV6b       F       MCTCLLCCVAFCLLQAGPV   NAGVIQTPKFQVLKM GQGVTLKCAQD LKH.......NY MYWYRQDP GHGLRLIHY SAG....SGT TNKGDIP.D GYSVSRS.DV ENFTLKLEYATP NQTSVYLC ASSY..[5.6.4]

TRBV7S1d     F       MGTRLLYWATLYLLGVGHT   EAGISQSPRHRVTGR GQNVTLRCDPV SDH.......NR LYWYRQTL GQGLEILTL FQN....KDA VDTSGMPDA RFSAKRP.QG LSSTLEIQRVEP GDSAVYLC ASSL..[5.6.4]
TRBV7S1f/b   P       MGTRLLYWATLCLLGVGHT   EAGISQSPRHRVTGR GQNVTLRCDPI SGH.......NS LF*YRQTL GQGLEILTL FQN....KDA VDTSGMPDA RFSAKRP.QG LSSTLEIQRVEP GDSAVYLC ASSL..[5.6.4]
TRBV7S2d     F       MGTRLLYWATLCLLGVGHT   KAGVSQSPRHRVTGR GQNVTLMCDPV SDH.......NR LYWYRQML GQGLEILTY FQD....DDA LDKSGMPDA RFSAERP.QG LSSTLKIQRVEP GDSAEYLC ASSL..[5.6.4]
TRBV7S2f/b   F       MGTRLLYWATLCLLGVGHT   KAGVSQSPRHRVTGR GQNVTLRCDPV SDH.......NR LYWYRQML GQGLEILTY FQD....DDA LDKSGMPDA RFSAERP.QG LSSTLKIQRVEP GDSAEYLC ASSL..[5.6.4]

TRBV8d       F        MSPSLFCVILCLLGAGPT   DAEITQTPRYRIVST GHKTVLECSQH MNH.......FG MFWYQQDP GQGPGLIHY SND....IGS TAEGEVT.E GYRVSRP.EK AHFPLTLESAST NQTSLYLC ASSV..[5.6.4]
TRBV8f       F        MSPSLFCVILCLLGAGPT   EAEITQTPRYRIVST GHKTVLECSQH MNH.......FG MFWYRQDP GQGPRLIHY SND....IGS TAEGEVT.E GYRVSRP.EK AHFPLTLESAST NQTSLYLC ASSV..[5.6.4]
TRBV8b       F        MSPSLFCVILCLLGAGPT   DAEITQTPRYRIVST GHKTVLECSQH MNH.......FG MFWYQQDP GQGPRLIHY SND....IGS TAEGEVT.E GYRVSRP.EK AHFPLTLESAST NQTSLYLC ASSV..[5.6.4]

TRBV10d/f/b  F       MVTRLFFCAASCLLWTGQV   EAGITQSPRYKIAVI GETLTLQCHQT DNH.......DY MYWYRQDL GHGLKLIYY TYD....VNK TDKGEIP.D GYSVSRS.NI GDFPLTLESVVP SRTSAYLC ASSN..[5.6.4]

TRBV11d/f/b  F       MGTRLLCWATLCLLGAELT   EGGVAQSPRHKITEE RQAVALWCDPI SGH.......SF LYWYRQTL EQDLELLIL FEE....TAG INVLQSPKN RFSAERP.SG ANSTLKIQSAEL GDSAVYLC ASSL..[5.6.4]

TRBV12S1f    P       MGPRALCCVALCLLGVEPT   DAGVTQTRRHQVAGM GQAVTLECEPV SDH.......TA LFWYRQTS EQGLALLIY FRN....QAP VDDTGMPKD RFLAKMT.NE SLHPEYPAGRTQ GLGHVPLC QQRG..[5.6.4]
TRBV12S2f    F          MAARLLCCVALCLLGV   DAGVTQTPRHQVAGM GQAVTLGCEPA SGH.......AA LFWYRQTS EQGLALLIY FNN....QDP VDDTGMPKD RFLATMT.NK SFSTLNIQPAEP RDSATYLC ASSM..[5.6.4]

TRBV14f      F       MVSRLLSLVSLCLLGARHL   LAGVAQFPSHRVIEK KQAVSVRCDPV SGH.......EY LVWYQHAM GREMKFLMS FLR....DTV RDASGMLKD RFSAERT.EG TYSTLKVHPAEP EDSGVYFC ASSL..[5.6.4]
TRBV14b      F       MVSRLLSLVSFCLLGARHL   LAGVAQFPSHRVIEK KQAVSVRCDPV SGH.......EY LVWYQHAV GREMKFLMS FLR....DTV RDASGMLKD RFSAERT.EG TYSTLKVHPAEP EDSGVYFC ASSL..[5.6.4]

TRBV15S1d    F       MGPRLLHCVALCLFGAGPV   GAMVNQTPRYWVTGT GKPVTLNCSQT LNH.......DT MYWYQQKP SQAPKQLLY YYN....TQL NRETDTS.D NFQSRQF.NT SSCSLDIRSAGV GDSAVYLC ASSR..[5.6.4]
TRBV15S2d    F       MGPRLLHCVALCLFGAGPV   GAMVNQTPRYWVTGT GKPVTLNCSQT LNH.......DT MYWYQQKP SQAPKQLLY YYN....TQL NRETDTS.D NFQSRQF.NT SSCSLDIRSAGV GDSAVYLC ASSR..[5.6.4]
TRBV15f      F       MGPRLLHCVALCLLGAGPV   GAMVNQTPRYWVTGT GKPVTLNCSQT LNH.......DT MYWYQQKP SQAPKQLLY YYN....TQL NRETDTS.D NFQSRQF.NT SSCSLDIRSAGV GDSAVYLC ASSR..[5.6.4]
TRBV15b      F       MGPRLLHCVALCLLGAGPV   GAMVNQTPRYWVTGT GKPVTLNCSQT LNH.......DT MYWYQQKP SQAPKLLLY YYI....TQL NRETDTS.D NFQSRQF.NT SSCSLDIRSAGV GDSAMYLC ASSR..[5.6.4]

TRBV16d      F       MCQTVLGLMVLCLLGAGFL   EAEVTQTPGHLVKGT GQKAEMYCVPR KGH.......NY VCWYQQIP AKEFKFLIS FQN....NNE LDKTGMPKE RFSAVCY.QN SSCTLKIQPAAL QDSAVYLC ASSE..[5.6.4]
TRBV16f      P       MCQTVLGLMVLCLLGAGFL   EAEVTQTPGHLVKGT GQKAEMYCVPR KGH.......NY V*WYQQIP AKEFKFLIS FQN....NNE LDKTGMPKE RFSAVCY.QN SSCTLKIQPAAL QDSAVYLC ASSE..[5.6.4]

TRBV19d      F       MGSSVIYWVALCLIGAGTV   DGGITQTPKYVLKEE GRDVTLECEQD FNY.......DT MYWYRQDP GQRPRLIYF SLS....AKD VQKGDMA.E GYSASRE.KK PLFPLTVTLTQK NQTAVYLC ASSI..[5.6.4]
TRBV19f/b    F       MGSPVIYWVALCLLGAGTV   DGGITQTPKYVLKEE GRDVTLECEQD FNY.......DT MYWYRQDP GQRPRLIYF SLS....AKD VQKGDMA.E GYSASRE.KK PLFPLTVTLTQK NQTAVYLC ASSI..[5.6.4]

TRBV20d      F            MLLFLLLLGPGSGL   GAQVSQHPSRAIRER GASVTFQCRAE DFQ......ATI MFWYRQFP EQGLTLIAT SNQG...SNA TYEQGFTKD KFPINHP.SL TFSSLTVTSVSP ADSSLYFC SAS...[6.7.3]
TRBV20b      nd    ----------------SGL   GAQVSQHPSRAIRER GASVTFQCRAE DFQ......ATI MFWYRQFP EQGLTLIAT SNQG...SNA TYEQGFTKD KFPINHP.SL TFSSLTVTSVSP ADSSLYFC SAS...[6.7.3]

TRBV21S1d    F       MCQGLLCCLALLFWGAGST   DTGVTQSPGHLVREK EQRAKMDCVPK EGH.......NY VYWYRKKL EEAFEFLVY LRN....GGD IMEEAEGFK QRFSAKCPKN SPCSLEINSTQA GDSARYFC ASSE..[5.6.4]
TRBV21S1b    F       MCLSLLCCVALLLWGAGST   DTGVTQSPGHLVREK EQRAKMDCVPK EGH.......NY VYWYRKKL EEAFEFLVY LRN....GGD IMEEAEGFK QRFSAKCPKN SPCSLEINSTQA GDSARYFC ASSE..[5.6.4]
TRBV21S2d/b  F       MCLSLLCCVALLLWGAGSV   DTGVTQSPGHLVREK EQRAKMYCVPK EGH.......AY VYWYRKKL EEAFEFLVY LRN....EDI VEKIAGFEQ RYSAKCP.KN SPCSLEINSTQA GDSARYFC ASSE..[5.6.4]
TRBV21f      F       MCFSLLCCVALLLWGAGSV   DTGVTQSPGHLVRGK EQRAKMHCVPI KGH.......TL VFWYRKKL EEAFEFLVY LRN....EDI VDKIAGFEQ RYSAKCP.KN SPCSLEINSTQA GDSARYFC ASSE..[5.6.4]

TRBV22d/f    F        MCSWALCYVVLCLLGAGP   TDAEIYQRRFLLAGA GRDVTLECEQS LRY.......NA MYWYRQDP GLGLRLIYY SKV....EKD VQRGDIS.E GYSVSRE.EK GLFPLTVKLAHI NQTALYVC SGSA..[5.6.4]
TRBV22b      F        MCSWALCYVALCLLGAGP   TDAEIYQRRFLLAGA GRGVTLECKQS LRY.......NA MFWYPQDL GLGLRLIYY STV....EKD VQRGDIS.E GYSVSRE.EK GLFPLTVKLAHI NQTALYVC SGSA..[5.6.4]

TRBV24f      F       MGSMLLCCVAFCLLGTGSM   DTGVTQTPRNRITNT GNSVLLECFQT KGH.......NY MYWYRQDP GLGLRLIYS SYN....VND TNKGELS.N GYRVSRA.EK AKFFLFLETPVP NQTALYFC ASSYL.[5.6.5]
TRBV24b      F       MGSMLLCCVAFCLLGTGSM   DTGVTQTPRNRITNT GNSVLLECSQT KGH.......DY MYWYRQDP GLGLRLIYS SYN....VND TNKGELS.N GYRVSRM.EK EKFFLFLETPVP NQTALYFC ASSYL.[5.6.5]

TRBV25d/f    F       MAVRLLCYVALSLLGAGLT   NADVNQTPRYAVIGT GKKITLECNQA MDH.......ES MYWYRQDP GMEPQLIHY SHG....VNT TEKGDRP.S ESTVSRI.RK DQFPLTLEATSP SQTSRYFC ASSE..[5.6.4]
TRBV25b      F       MAVRLLCYVALSLLGAGLT   NADVNQTPRYAVIGT GKKITLECNQA MDH.......ES MYWYRQDP GMEPQLIHY SRG....VNT TEKGDRP.S ESTVSRI.RK DQFPLTLEATSP SQTSTYFC ASSE..[5.6.4]

TRBV26d/f/b  F       MSNRLLCCALIFLIRAGLK   EAAVTQSPRHRILKT EQKLTLQCSQR MNH.......YS MYWYRQDP GFGLRLIYY STG....TRT TKQGDVP.E GYHVSRE.DQ ADFPLTLQSAST NQTSVYFC ASSE..[5.6.4]

TRBV27d      F       MGPRLLGWVLLWLLGAGPV   EADVTQSPTHRITET RKKLTVTCSQN MNH.......EA MYWYRQDP GLGLRLIYF SRN....VNF TEKGDIP.D GYTVSRK.EK KDFSLTLESASI TQTSLYLC ASSV..[5.6.4]
TRBV27f      F       MGPRLLGWVLLWLLGAGPV   EPDVTQSPTHRITET RKKLTVTCSQN MNH.......EA MYWYRQDP GLGLRLIYF SRN....VNF TEKGDIP.D GYTVSRK.EK KDFSLTLESASI TQTSLYLC ASSV..[5.6.4]
TRBV27b      F       MGPRLLGWVLLWLLGAGPV   EADVTQSPTHCITET RKKLTVTCSQN MNH.......DA MYWYRQDP GLGLRLIYF SRN....VNF TEKGDIP.D GYTVSRK.EK KDFSLTLESASI TQTSLYLC ASSV..[5.6.4]

TRBV28d      F         MGIRLLCAFCFLGVGLM   DAKVTQIPRYLVKRM GEKVLIDCVQD MGH.......ER MFWYRQDP ALGLQLLHY SYD....VDI VEKGDMP.E GYNVSRK.KK ERFALTLESAST SQTSVYLC ASSI..[5.6.4]
TRBV28b      F         MGIRLLCAFCFLGVGLM   DAKVTQIPRYLVKRM GEKVLIDCVQD MGH.......ER MFWYRQDS ALGLRLLHY SYD....VDI VEKGDMP.E GYNVSRK.KK ERFALTLESAST SQTSVYLC ASSI..[5.6.4]

TRBV29d/f/b  F           MLTLLLLLLGLGSAF   GALVSQKPSRAICQH GVSVMIQCEAD SQV.......IS MYWYRQRP GQSLSLIAT ANQG...SQA TYESGFAKE KFPISRP.SL NFSVLTVSSVSP EDSSSYFC SA....[5.7.2]

TRBV30d/f/b  F         MPCCLLALLLGTFLGVR   AQTIHQRPPTMVQPM GSSLSLECTVK GTS......NPS LYWYRQAA GGALQLLFY SFS.....AD QVDSEEP.Q NFEASVS.QD GSFILSSKKLLL NNSGFYLC AWS...[6.5.3]
```

(a)

```
  TRBD                  5'D-NONAMER   5'D-SPACER 5'D-HEPTAMER                        3'D-HEPTAMER    3'D-SPACER             3'D-NONAMER
 gene name               GGTTTTTGT    ************  CACTGTG         D-REGION             CACAGTG   **********************   ACAAAAACC

TRBD1 Cambac/ Camfer     cgtttttgt    ataaagctgtaa  cgttgtg        GGGACAGGGGGC          cacggtg   attcaaccctatgggaaatcttt  acaaaaacc
                                                                   G  T  G  G
                                                                    G  Q  G
                                                                     D  R  G

TRBD3 Cambac             catttttgt    atcctggtgtaa  cattgtg    GGGACTATAGCGGGGGGAGG      cacaatg   attcaggtagagaaggtcctttt  acaaaaagc
                                                               G  T  I  A  G  G
                                                                G  L  *  R  G  E
                                                                 D  Y  S  G  G  R
TRBD2 Cambac             catttttgt    atcttggcttaa  cattgtg      GGACTATGGGGGGAG          cacaatg   attcaggtagagaaggtcctttt  acaaaaagc
                                                                  G  L  W  G  E
                                                                   D  Y  G  G
                                                                    T  M  G  G
```

(b)

```
  TRBJ                    J-NONAMER     J-SPACER    J-HEPTAMER
 gene name                GGTTTTTGT   ************   CACTGTG                    J-REGION                         5'splice donor

TRBJ1-1 Cambac/Camfer     gttttcctt   tttgctccgtgt   cactgtg      TGAACACTGAAGTTTTCTTTGGAGAAGGCACCAGACTCACGGTTGTAG     gtaaga
                                                                    N  T  E  V  F  F  G  E  G  T  R  L  T  V  V
TRBJ1-2 Cambac/Camfer     cgttgtagg   gtggctgtattc   tgatgtg          TTATGACTTTAACTTCGGCCCAGGGACCAAGCTGACGGTCGTAG     gtaagg
                                                                          Y  D  F  N  F  G  P  G  T  K  L  T  V  V
TRBJ1-3 Cambac/Camfer     ggttttgaa   gtggatctggga   ggctgtg    CTTTACAGACACCTACTATTTTGGGGAGGGAAGCCGGCTCACTGTTGTAG     gtaagc
                                                                 F  T  D  T  Y  Y  F  G  E  G  S  R  L  T  V  V
TRBJ1-4 Cambac/Camfer     agttttcct   accaggctttag   tgttgtg   TGACTAATGAAAAACTCTATTTCGGCAGCGGGACGAAGCTTTCCGTCTTGG    gtaagt
                                                                 T  N  E  K  L  Y  F  G  S  G  T  K  L  S  V  L
TRBJ1-5 Cambac/Camfer     gggtctgcc   acactcgtgtgt   tactgtg    TAGCAACCGGGTGCAGCATTTTGGAGAAGGGACTCGACTCTCTGTCCTAG     gtaagg
                                                                 S  N  R  V  Q  H  F  G  E  G  T  R  L  S  V  L
TRBJ1-6 Cambac            ggttttacc   acagctgtctgc   agctgtg TTCCTATAATTCACCCCTCCACTTTGGGCTCAGCACCAGGCTCACCGTGACAG     gtatgg
                                                              S  Y  N  S  P  L  H  F  G  L  S  T  R  L  T  V  T
TRBJ1-6 Camfer            ggttttacc   acagctgtctgc   agctgtg TTCCTATAATTCACCCCTCCACTTTGGGCTCGGCACCAGGCTCACCGTGACAG     gtatgg
                                                              S  Y  N  S  P  L  H  F  G  L  G  T  R  L  T  V  T
TRBJ1-7 Camfer            agttttcct   accaggctttag   tgttgtg   TGACTAATGAAAAACTCTATTTCGGCAGCGGGACGAAGCTTTCCGTCTTGG     gtaagt
                                                                 T  N  E  K  L  Y  F  G  S  G  T  K  L  S  V  L
TRBJ3-1 Cambac            gaattcttg   gcagccccttct   cactgtg    CTCCTACGCTGAGCAGTACTTCGGAGCAGGGACTCGGCTCACTGTGCTAG     gtaaga
                                                                 S  Y  A  E  Q  Y  F  G  A  G  T  R  L  T  V  L
TRBJ3-2 Cambac            agtttgctt   gtggatctccag   ggctgtg   TAAACAGCCAGCAGCTGTACTTTGGGGAGGGTTCCAAGCTGATTGTCCTGG     gtaagg
                                                                 N  S  Q  Q  L  Y  F  G  E  G  S  K  L  I  V  L
TRBJ3-3 Cambac            ggtttttgg   cctgggttccag   ggctgtg     AGCACAGATCCTCTGTATTTCGGCGGGGGCACCCGTCTGACCGTGCTAG     gtaagc
                                                                 S  T  D  P  L  Y  F  G  G  G  T  R  L  T  V  L
TRBJ3-4 Cambac            tgtttttgt   gctgcgccccgg   ggctgtg     AGCCAGAACACTCAGTACTTTGGCGCAGGCACCCGGCTGTCGGTGCTAG     gtgagc
                                                                 S  Q  N  T  Q  Y  F  G  A  G  T  R  L  S  V  L
TRBJ3-5 Cambac            ggtttttgc   gcggggctgggg   ggccgtg      ACTCACAGACCCAGTACTTCGGGCCGGGCACGCGGCTCCTGG------     ------
                                                                    S  Q  T  Q  Y  F  G  P  G  T  R  L  L
TRBJ3-7 Cambac            tgtgtgcgt   gcggggctcctc   ctctgtg       GTCCTATGAGCAGCATTTCGGCCCAGGCACCAGGCTCACGGTCTTAG     gtaaga
                                                                    S  Y  E  Q  H  F  G  P  G  T  R  L  T  V  L
TRBJ2-1 Cambac            gaattcttg   gcagccccttct   cactgtg    CTCCTACGCTGAGCAGTACTTCGGAGCAGGGACTCGGCTCACTGTGCTAG     gtaaga
                                                                 S  Y  A  E  Q  Y  F  G  A  G  T  R  L  T  V  L
TRBJ2-2 Cambac            agtcggtgc   cttagtccccag   ggctgtg   TGAACACCGGACAGCTGTACTTTGGGGAAGGTTCCAAGCTGACTGTGCCGG     gtaagg
                                                                 N  T  G  Q  L  Y  F  G  E  G  S  K  L  T  V  P
TRBJ2-3 Cambac            gggtttttg   tcctgggcctcg   ggctgtg         AGCAGACACTGTTTTTCGGCGGGGGAACCAGGCTGTCTGTGCTAG     gtgagc
                                                                       Q  T  L  F  F  G  G  G  T  R  L  S  V  L
TRBJ2-4 Cambac            ggtttctgt   gcggggttgggg   ggctgtg      AACAAGAGACCCAGTACTTCGGGCCGGGCACGCGGCTCCTGGTGCTAG     gtgagt
                                                                    Q  E  T  Q  Y  F  G  P  G  T  R  L  L  V  L
TRBJ2-5 Cambac            ggtttctgg   ggggagcccggg   ggctgtg  CTTCCAGCAGCCGCCCTGACCTTCGGGGCCGGCAGCCGGCTGGCCGTGCCGG     gtgagt
                                                              L  P  A  A  A  L  T  F  G  A  G  S  R  L  A  V  P
TRBJ2-6 Cambac            ---ttgcgt   gcggggctcctc   ctttgtg       CTCCTATGAGAGGTATTTCGGCCCAGGCACCAGGCTCACGGTCTTAG     gtaaga
                                                                    S  Y  E  R  Y  F  G  P  G  T  R  L  T  V  L
```

(c)

```
                                  A        AB      B          BC         C     CD      D             DE              E       EF   F              FG                    G
                               (1-15)           (16-26)    (27-38)    (39-45)       (77-84)                       (85-96)       (97-104)        (105-117)            (118-128)
                          ----------->      ---------->             -------->      --------->                   ---------->    -------->                              --------->
                           1       10   15  16      2326 27      36  3941  45       77 80 84               85  89      96  97     104 105                117   118121   128
                   87654321|........|....|123|......|..| |........| |.|...|1234567|..|...|12345677654321|...|......|12|......| |......123456654321.....|  |..|......|
TRBC1  Camdro      (E)DLSQVKPPKVVVFEPSEAEISR..TQKATLVCLAT.GFYP..DHVE LSWWVNGKEVQS..GVSADPQPYREDPNL.SDSTYCLSSRLRVSATFWH.NPRNHFRC QVHFHGLTDKDRDQWKEGWPMPISQNV SAETWGRA..
TRBC1  Cambac      ----------------------------------------------------------------------------------------------------------QVHFHGLTDKDRDQWKEDWPMPVSQNV SAEAWGRA..

TRBC3  Camdro      (E)DLSQVKPPKVVVFEPSEAEISR..TQKATLVCLAT.GFYP..DHVE LSWWVNGKEVQS..GVSADPQPYREDPNL.SDSTYCLSSRLRVSATFWH.NPRNHFRC QVHFHGLTDKDRDQWKEGWPMPISQNV SAETWGRA..
TRBC3  Cambac      (E)DLSQVKPPKVAVFEPSEAEISR..TQKATLVCLAT.GFYP..DHVE LSWWVNGKEVQS..GVSTDPQPYREDPSL.SDSTYCLSSRLRVSATFWH.NPRNHFRC QVHFHGLTDKDRDQWKEDWPLPVSQNV SAEAWGRA..

TRBC2  Camdro      (E)DLSQVKPPKVAVFEPSEAEISR..TQKATLVCLAT.GFYP..DHVE LSWWVNGKEVQS..GVSTDPQPYREDPSL.SDSTYCLSSRLRVSATFWH.NPRNHFRC QVHFHGLTDKDRDQWKEDWPMPVSQNV SAEAWGRA..
TRBC2  Cambac      (E)DLSQVKPPKVAVFEPSEAEISR..TQKATLVCLAT.GFYP..DHVE LSWWVNGKEVQS..GVSTDPQPYREDPSL.SDSTYCLSSRLRVSATFWH.NPRNHFRC QVHFHGLTDKDRDQWKEDWPMPVSQNV SAEAWGRA..
TRBC2  Camfer      (E)DLSQVKPPKVAVFEPSEAEISR..TQKATLVCLAT.GFYP..DHVE LSWWVNGKEVQS..GVSTDPQPYREDPSL.SDSTYCLSSRLRVSATFWH.NPRNHFRC.QVHFHGLTDKDRDQWKEGWPMPVSQNV SAEAWGRA..
```

```
                   CONNECTING-REGION    | TRANSMEMBRANE-REGION |CYTOPLASMIC-REGION

                      [EX2]                   [EX3]                    [EX4]
TRBC1  Camdro      (H)CGFTS  (V)SYQQGVLSATLLYEILLGKATLYAVLVSALVLMAM  VKRKDS
TRBC1  Cambac      (D)CGFTS  (V)SYQQGVLSATLLYEILLGKATLYAVLVSALVLMAM  VKRKDS

TRBC3  Camdro      (H)CGFTS  (V)SYQQGVLSATLLYEILLGKATLYAVLVSALVLMAM  VKRKDS
TRBC3  Cambac      (D)CGFTS  (V)SYQQGVLSATLLYEILLGKATLYAVLVSALVLMAM  VKRKDS

TRBC2  Camdro      (D)CGFTS  (V)SYQQGVLSATLLYEILLGKATLYAVLVSALVLMAM  VKRKDS
TRBC2  Cambac      (D)CGFTS  (V)SYQQGVLSATLLYEILLGKATLYAVLVSALVLMAM  VKKKDS
TRBC2  Camfer      (D)CGFTS  (V)SYQQGVLSATLLYEILLGKATLYAVLVSALVLMAM  VKKKDS
```

Supplementary Figure S3

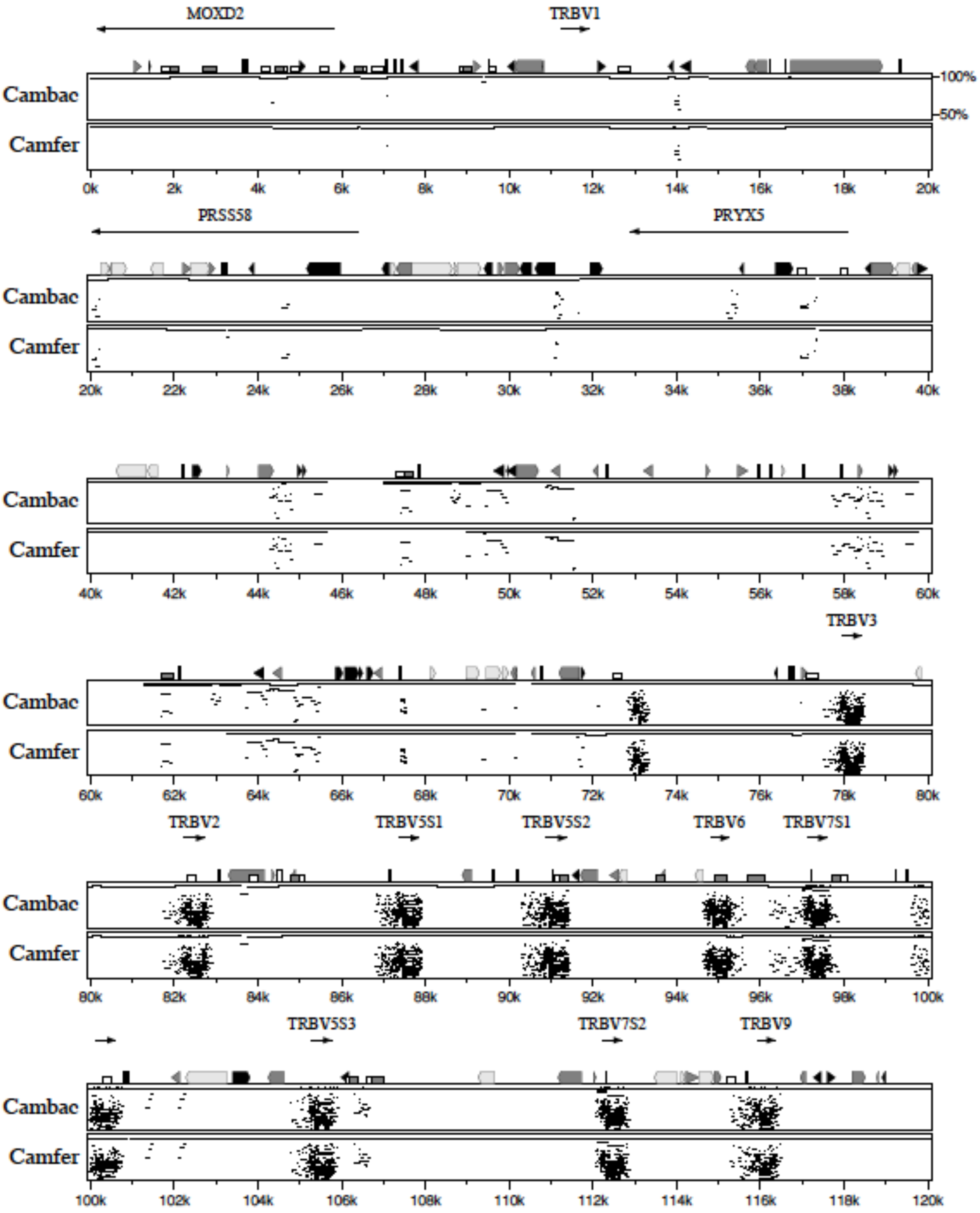

MOXD2
TRBV1
Cambac
Camfer
100%
50%
0k
2k
4k
6k
8k
10k
12k
14k
16k
18k
20k
PRSS58
PRYX5
22k
24k
26k
28k
30k
32k
34k
36k
38k
40k
42k
44k
46k
48k
50k
52k
54k
56k
58k
60k
TRBV3
62k
64k
66k
68k
70k
72k
74k
76k
78k
80k
TRBV2
TRBV5S1
TRBV5S2
TRBV6
TRBV7S1
82k
84k
86k
88k
90k
92k
94k
96k
98k
100k
TRBV5S3
TRBV7S2
TRBV9
102k
104k
106k
108k
110k
112k
114k
116k
118k
120k

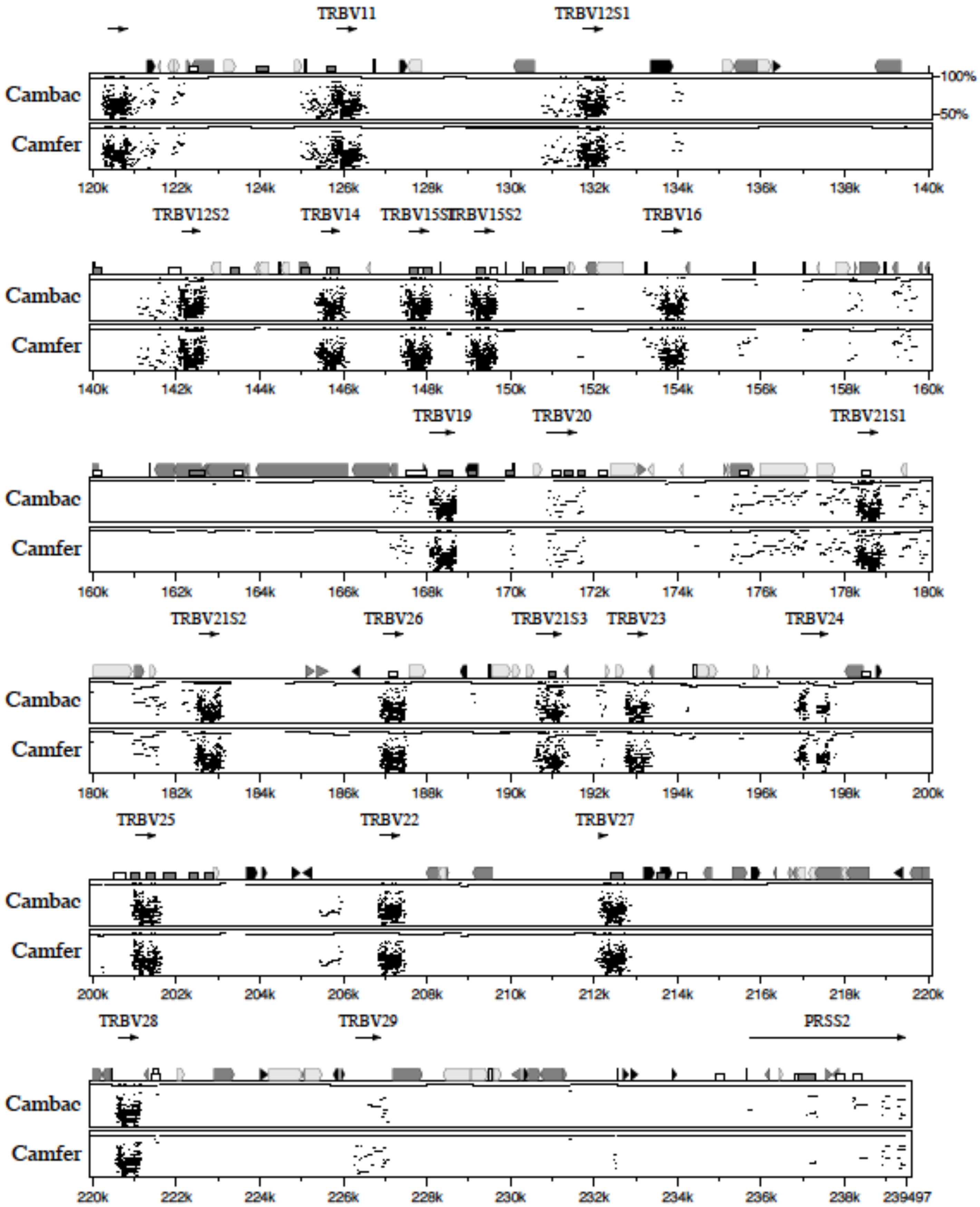
TRBV11
TRBV12S1
Cambac
Camfer
100%
50%
120k 122k 124k 126k 128k 130k 132k 134k 136k 138k 140k
TRBV12S2
TRBV14
TRBV15S1
TRBV15S2
TRBV16
140k 142k 144k 146k 148k 150k 152k 154k 156k 158k 160k
TRBV19
TRBV20
TRBV21S1
160k 162k 164k 166k 168k 170k 172k 174k 176k 178k 180k
TRBV21S2
TRBV26
TRBV21S3
TRBV23
TRBV24
180k 182k 184k 186k 188k 190k 192k 194k 196k 198k 200k
TRBV25
TRBV22
TRBV27
200k 202k 204k 206k 208k 210k 212k 214k 216k 218k 220k
TRBV28
TRBV29
PRSS2
220k 222k 224k 226k 228k 230k 232k 234k 236k 238k 239497

Legend

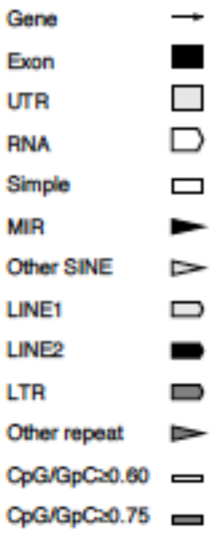

Supplementary Figure S4

Supplementary Table S1. Scaffolds of the camel genomic assemblies used in the analysis.

Species	**Assembly**	**Scaffolds (bp)**
Camelus ferus	CB1	NW_006210980 (302258)
Camelus bactrianus	Ca_bactrianus_MBC_1.0	NW_011511605 (181382)<br>NW_011509864 (99628)<br>NW_011537499 (542)<br>NW_011541550 (877)<br>NW_011529568 (303)<br>NW_011514083 (2181)<br>NW_011511596 (685)

Supplementary Table S2. Description of the TRB genes in the *Camelus ferus* genome assembly. The position of all genes and their classification and functionality are reported.

**Gene classification**	**Functionality**	**Position[a] in NW_006210980**
TRBV1	P	2814238-2814861
TRBV2	F	2883236-2883687
TRBV3	P	2878903-2879363
TRBV5S1	F	2888154-2888613
TRBV5S2	F	2891651-2892130
TRBV5S3	F	2906767-2907222
TRBV6	F	2895581-2896012
TRBV7S1	P	2898019-2898476
TRBV7S2	F	2913787-2914243
TRBV8	F	2901064-2901584
TRBV9	P	2917482-2917906
TRBV10	F	2921943-2922385
TRBV11	F	2927274-292722
TRBV12S1	P	2933182-2933620
TRBV12S2	F	2943582-2944029
TRBV14	F	2946924-2947359
TRBV15	F	2949022-2949476
TRBV16	P	2953880-2954333
TRBV19	F	2967958-2968436
TRBV20	ORF	2970037-2970744
TRBV21	F	2982817-2983273
TRBV22	F	2987405-2987910
TRBV23	P	2991431-2991882
TRBV24	F	2998293-2998341
TRBV25	F	3003467-3003937
TRBV26	P	3009664-3010143
TRBV27	F	3015185-3015653
TRBV28	P	3023553-3024007
TRBV29	F	3029577-3030179
TRBV30	F	3065530-3064848
TRBD1	F	3047512-3047523
TRBJ1-1	F	3048161-3048208
TRBJ1-2	F	3048296-3048339
TRBJ1-3	F	3048545-3048594
TRBJ1-4	F	3049146-3049196
TRBJ1-5	F	3049423-3049472
TRBJ1-6	F	3049909-3049961
TRBJ1-7	*F*	*3052256-3052306*
TRBC2	F	3052730-3054235

[a] L-PART1/ V-exon for TRBV genes and coding sequence for TRBD and TRBJ

Supplementary Table S3. Description of the TRB genes in the *Camelus bactrianus* genome assembly. The position of all genes and their classification and functionality are reported.

Gene classification	Functionality[a]	NCBI Reference Sequence	Position[b]
TRBV1	F	NW_011511605	169395-169120
TRBV2	F	NW_011511605	98573-98467
TRBV3	P	NW_011511605	102909-102791
TRBV5S1	F	NW_011511605	93668-93550
TRBV5S2	F	NW_011511605	90221-90084
TRBV5S3	F	NW_011511605	75841-75727
TRBV6	F	NW_011511605	86291-86205
TRBV7S1	P	NW_011511605	83968-83872
TRBV7S2	F	NW_011511605	68907-68791
TRBV8	F	NW_011511605	80976-80845
TRBV9	P	NW_011511605	65215-65119
TRBV10	F	NW_011511605	60752-60653
TRBV11	F	NW_011511605	55505-55402
TRBV12S1	P	NW_011511605	49635-49518
TRBV12S2	P	NW_011511605	39280-39259
TRBV14	F	NW_011511605	35990-35900
TRBV15S1	F	NW_011511605	33894-33782
TRBV15S2	nd	NW_011541550<br>NW_011529568	587-877<br>1-82
TRBV16	F	NW_011514083	1046-1500
TRBV19	F	NW_011511605	15711-15575
TRBV20	nd	NW_011511605	12498 -12801
TRBV21S1	F	NW_011511605	5236-5124
TRBV21S2	F	NW_011509864	1558179-1558067
TRBV21S3	nd	NW_011511596	323-685
TRBV22	F	NW_011509864	1553603-1553472
TRBV23	P	NW_011509864	1549807-1549701
TRBV24	F	NW_011509864	1543346-1543217
TRBV25	F	NW_011509864	1538342-1538214
TRBV26	F	NW_011509864	1532247-1532110
TRBV27	F	NW_011509864	1526887-1526761
TRBV28	F	NW_011509864	1518537-1518415
TRBV29	F	NW_011509864	1512913-1512641
TRBV30	F	NW_011509864	1459915-1460264
TRBD1	F	NW_011509864	1495715-1495726
TRBJ1-1	F	NW_011509864	1495030-1495077
TRBJ1-2	F	NW_011509864	1494899-1494942
TRBJ1-3	F	NW_011509864	1494644-1494693
TRBJ1-4	F	NW_011509864	1494042-1494092
TRBJ1-5	F	NW_011509864	1493766-1493815
TRBJ1-6	F	NW_011509864	1493275-1493327
TRBC1	P	NW_011509864	1480663-1491092
TRBD3	F	NW_011509864	1487034-1487015
TRBJ3-1	F	NW_011509864	1475828-1475877
TRBJ3-2	F	NW_011509864	1486121-1486171
TRBJ3-3	F	NW_011509864	1485907-1485955
TRBJ3-4	F	NW_011509864	1485762-1485810
TRBJ3-5	nd	NW_011509864	1485706-1485665
TRBJ3-7	F	NW_011509864	1485381-1485427
TRBC3	F	NW_011509864	1481703-1480663
TRBD2	F	NW_011509864	1476529-1476543
TRBJ2-1	F	NW_011509864	1475828-1475877
TRBJ2-2	F	NW_011509864	1475632-1475682
TRBJ2-3	F	NW_011509864	1475420-1475464
TRBJ2-4	F	NW_011509864	1475305-1475352
TRBJ2-5	F	NW_011509864	1475203-1475254
TRBJ2-6	nd	NW_011509864	1475046-1475092
TRBC2	F	NW_011509864	1472235-1471194

[a]nd: not defined (indicates that the nt sequence of the gene is incomplete and its functionality cannot be defined).

[b] L-PART1/ V-exon for TRBV genes and coding sequence for TRBD and TRBJ.

Supplementary Table S4. Description of the unrelated TRB genes in the *Camelus ferus* genome. The position of all genes and their classification are also reported.

Gene classification	Functionality	NCBI Reference Sequence	Position
MOXD2	F	NW_006210980	2809092-2803163
TRY1	F	NW_006210980	2829206-2823099
TRY2	F	NW_006210980	2840842-2835973
TRY5	F	NW_006210980	3039075-3043104
EPBH6	F	NW_006210980	3104296-3111350

Supplementary Table S5. Description of the unrelated TRB genes in the *Camelus bactrianus* genome. The position of all genes and their classification are also reported.

Gene classification	Functionality	NCBI Reference Sequence	Position
MOXD2	F	NW_011511605	173902-180382
TRY1	F	NW_011511605	154437-160737
TRY2	F	NW_011511605	142898-147952
TRY3	F	NW_011511605	131685-135618
TRY4	F	NW_011511605	117332-121258
TRY5	F	NW_011509864	1503500-1499760
EPHB6	F	NW_011509864	1459085-1414085

Supplementary Table S6. GEDI ID of the genes used in the phylogenetic analysis.

Gene	Species	Accession number	Reference
TRBV	*Homo sapiens*	NG_001333	http://www.imgt.org/
	Camelus dromedarius	NW_011591622<br>NW_011593440<br>NW_011591151<br>NW_011620189<br>NW_011616084<br>NW_011607149<br>NW_011601111<br>LT837971	Antonacci et al., 2017a-b
	Camelus ferus	NW_006210980	this study
	Camelus bactrianus	NW_011511605<br>NW_011509864<br>NW_011514083<br>NW_011511596	this study
TRBC	*Homo sapiens*	NG_001333	http://www.imgt.org/
	Mus musculus	AE000665	http://www.imgt.org/
	Canis lupus familiaris	NW_003726086	Mineccia et al., 2012
	Oryctolagus cuniculus	NW_003159384	Antonacci et al., 2014
	Equus caballus	L27845<br>L27844	Schrenzel et al., 1994
	Ovis aries	AM420900	Antonacci et al., 2008
	Bos taurus	GK000004	Connelley et al., 2009
	Sus scrofa	NC_010460	Massari et al., 2018
	Camelus dromedarius	LT837971	Antonacci et al., 2017a
	Camelus ferus	NW_006210980	this study
	Camelus bactrianus	NW_011509864	this study
TRY	*Homo sapiens*	NG_001333	http://www.imgt.org/
	Canis lupus familiaris	NW_003726086	Mineccia et al., 2012
	Oryctolagus cuniculus	NW_003159384	Antonacci et al., 2014
	Bos taurus	GK000004	Connelley et al., 2009
	Ovis aries	NC_019461	personal communication
	Sus scrofa	NC_010460	Massari et al., 2018
	Camelus dromedarius	NW_011591622<br>NW_011591151	Antonacci et al., 2017a-b
	Camelus ferus	NW_006210980	this study
	Camelus bactrianus	NW_011511605<br>NW_011509864	this study

Supplementary Table S7. Description of *Camfer* and *Cambac* TRBV ORF and pseudogenes

TRBV genes	Defective Leader	Frameshift	Stop codon	Defective Vexon	Defective RSS
TRBV1*Camfer*	●				
TRBV3*Camfer*			●		
TRBV3*Cambac*			●		
TRBV7S1*Camfer*			●		
TRBV7S1*Cambac*			●		
TRBV9 *Camfer*	●				●
TRBV9 *Cambac*	●	●			●
TRBV12S1*Camfer*		●			
TRBV12S1*Cambac*		●			
TRBV12S2*Cambac*				●	
TRBV16 *Camfer*			●		
TRBV23 *Camfer*				●	
TRBV23 *Cambac*				●	
TRBV26 *Camfer*					●
TRBV28 *Camfer*				●	

Supplementary Table S8. Output file of the program RepeatMasker. Summary of the repeat content of *Camelus dromedarius*, *Camelus ferus* and *Camelus bactrianus* TRBV region is shown. The GC levels are also indicated.

```
==================================================
file name: Camdrom
total length:     239497 bp
GC level:         45.31 %
bases masked:      67412 bp ( 28.15 %)
==================================================
               number of      length   percentage
               elements*    occupied  of sequence
--------------------------------------------------
SINEs:                34         5622 bp    2.35 %
      Alu/B1           0            0 bp    0.00 %
      MIRs            30         5265 bp    2.20 %

LINEs:                87        29423 bp   12.29 %
      LINE1           59        21481 bp    8.97 %
      LINE2           25         7352 bp    3.07 %
      L3/CR1           3          590 bp    0.25 %
      RTE              0            0 bp    0.00 %

LTR elements:         54        25917 bp   10.82 %
      ERVL            14         6961 bp    2.91 %
      ERVL-MaLRs      17         4599 bp    1.92 %
      ERV_classI      18        13929 bp    5.82 %
      ERV_classII      2          113 bp    0.05 %

DNA elements:         26         4247 bp    1.77 %
      hAT-Charlie     12         2301 bp    0.96 %
      TcMar-Tigger     3          605 bp    0.25 %

Unclassified:          0            0 bp    0.00 %

Total interspersed repeats:     65209 bp   27.23 %


Small RNA:             3          312 bp    0.13 %

Satellites:            1          117 bp    0.05 %
Simple repeats:       47         1777 bp    0.74 %
Low complexity:        8          309 bp    0.13 %
==================================================
```

```
==================================================
file name: Camfer
total length:     240340 bp
GC level:         45.14 %
bases masked:      71321 bp ( 29.68 %)
==================================================
               number of      length   percentage
               elements*    occupied  of sequence
--------------------------------------------------
SINEs:                38         6214 bp    2.59 %
      Alu/B1           0            0 bp    0.00 %
      MIRs            33         5766 bp    2.40 %

LINEs:                94        31752 bp   13.21 %
      LINE1           63        23747 bp    9.88 %
      LINE2           27         7419 bp    3.09 %
      L3/CR1           4          586 bp    0.24 %
      RTE              0            0 bp    0.00 %

LTR elements:         58        26981 bp   11.23 %
      ERVL            15         7114 bp    2.96 %
      ERVL-MaLRs      15         4520 bp    1.88 %
      ERV_classI      22        14854 bp    6.18 %
      ERV_classII      3          176 bp    0.07 %

DNA elements:         27         4222 bp    1.76 %
      hAT-Charlie     13         2418 bp    1.01 %
      TcMar-Tigger     3          711 bp    0.30 %

Unclassified:          0            0 bp    0.00 %

Total interspersed repeats:     69169 bp   28.78 %


Small RNA:             4          403 bp    0.17 %

Satellites:            1          117 bp    0.05 %
Simple repeats:       46         1719 bp    0.72 %
Low complexity:        8          316 bp    0.13 %
==================================================
```

```
==================================================
file name: Cambac
total length:     239962 bp
GC level:         45.11 %
bases masked:      69148 bp ( 28.82 %)
==================================================
               number of      length   percentage
               elements*    occupied  of sequence
--------------------------------------------------
SINEs:                36         6014 bp    2.51 %
      Alu/B1           0            0 bp    0.00 %
      MIRs            31         5535 bp    2.31 %

LINEs:                82        31087 bp   12.95 %
      LINE1           55        23269 bp    9.70 %
      LINE2           24         7228 bp    3.01 %
      L3/CR1           3          590 bp    0.25 %
      RTE              0            0 bp    0.00 %

LTR elements:         52        26014 bp   10.84 %
      ERVL            14         6904 bp    2.88 %
      ERVL-MaLRs      14         4362 bp    1.82 %
      ERV_classI      18        14255 bp    5.94 %
      ERV_classII      3          176 bp    0.07 %

DNA elements:         26         4114 bp    1.71 %
      hAT-Charlie     13         2419 bp    1.01 %
      TcMar-Tigger     3          711 bp    0.30 %

Unclassified:          0            0 bp    0.00 %

Total interspersed repeats:     67229 bp   28.02 %


Small RNA:             4          434 bp    0.18 %

Satellites:            1          117 bp    0.05 %
Simple repeats:       43         1559 bp    0.65 %
Low complexity:        6          243 bp    0.10 %
==================================================
```

* most repeats fragmented by insertions or deletions have been counted as one element
The query species was assumed to be cetartiodactyla RepeatMasker version open-4.0.6, default mode
